# DNA Origami‐Templated Aptamer Chiral Structures Realize Cellular Enantioselectivity

**DOI:** 10.1002/adma.202519007

**Published:** 2026-01-16

**Authors:** Tingjie Song, Abhisek Dwivedy, Dhanush Gandavadi, Gayatri Chandran, Yiquan An, Mengxi Zheng, Xiaojing Wang, Lifeng Zhou, Yang Zhao, Xing Wang

**Affiliations:** ^1^ Carl R. Woese Institute for Genomic Biology University of Illinois at Urbana‐Champaign Urbana Illinois USA; ^2^ Holonyak Micro and Nanotechnology Lab Grainger College of Engineering University of Illinois at Urbana‐Champaign Urbana Illinois USA; ^3^ Department of Chemistry University of Illinois at Urbana‐Champaign Urbana Illinois USA; ^4^ Department of Bioengineering Grainger College of Engineering University of Illinois at Urbana‐Champaign Urbana Illinois USA; ^5^ Department of Electrical and Computer Engineering Grainger College of Engineering University of Illinois at Urbana‐Champaign Urbana Illinois USA; ^6^ Department of Advanced Manufacturing and Robotics Peking University Beijing China; ^7^ Cancer Center at Illinois University of Illinois at Urbana‐Champaign Urbana Illinois USA

**Keywords:** aptamer, cellular enantioselectivity, chirality, designer DNA nanostructure

## Abstract

Chirality refers to handedness as left‐ or right‐handed form of a molecule or molecular construct by synthesis or self‐assembly. In biology, chirality plays critical roles in determining the structures and functions of biomolecules such as proteins. In this study, we demonstrated that structural chirality generated by patterning multiple cell surface protein biomarker‐binding aptamers on a tubular‐shaped DNA origami (“DNA tube”) can realize differentness in cellular interaction, nanostructure uptake, cancer drug delivery, and consequent cell killing. Formation of the DNA tube and its ability to arrange external elements into left‐ or right‐handed form have been characterized by gel electrophoresis, atomic force microscopy, and transmission electron microscopy. Our fluorescence‐based cell experiments showed that the DNA tube‐templated aptamers of a left‐handed pattern can yield a higher cell uptake than those in a right‐form pattern. When loaded with an anticancer drug, Daunorubicin (Dau), the left‐handed tube‐aptamer‐Dau construct showed greater than double the cancer cell cytotoxicity than the construct in the right‐handed. Our study not only revealed that cell surface proteins interaction with the aptamers spatially organized into different chiral patterns can yield different cellular interaction and internalization efficiencies, but offered a new and versatile cancer drug delivery strategy for enhanced target cell treatment efficacy.

## Graphical abstract

Aptamers displayed on tubular DNA origami nanostructures in defined chiral orientations (left‐ or right‐handed) exhibit enantioselective interactions with cell surface target proteins. While the right‐handed configuration supports only transient binding, the left handedness energetically favors protein dimerization and promotes subsequent internalization of the origami‐aptamer‐protein complex. This enantioselective behavior may be leveraged for diverse therapeutic applications, including targeted drug delivery to cancer cells.

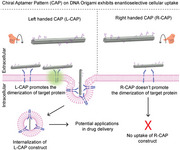

## 1 Introduction

Chiral patterns represent one of the central features in biological systems, as cells mostly consist of chiral molecules such as proteins and nucleic acids that build on smaller building blocks like amino acids and sugars. Often, chiral molecules or molecular constructs exhibit selective binding and interactions in one of the two forms. For example, the artificial sweetener aspartame, when bound in left‐handed (L‐) conformation to T1R2 receptor, exerts a sweet taste, while the right‐handed (R‐) conformation exerts a bitter taste [1]. Experimental modulation of helicity can result in biologically relevant functions, such as artificial D‐residue of naturally occurring α‐helices that create αL‐helical inhibitors of HIV fusion [2]. For instance, uniform chiral gold nanoparticles with intricate shapes can be synthesized using thiol‐containing chiral amino acids or peptides through seed‐mediated synthesis, in which introducing cysteine or glutathione to cubic seed nanoparticles induces opposing chiral properties [3]. Chiral assemblies with a gold shell and a core of gold nanoparticle satellites (linked via DNA hybridization) have been used to detect ochratoxin A (OTA) by measuring changes in circular dichroism signals [4]. Chiral gold nanorod assemblies can detect DNA at attomolar (am) concentrations using alternate polymerase chain reaction [5]. Chiral molybdenum oxide nanoparticles are effective in photothermal therapy, enhancing light‐induced heating under circularly polarized light, allowing for localized heating of tumor tissue without damaging surrounding healthy cells [6]. Additionally, gold nanobipyramids conjugated with D‐Glu demonstrate improved bactericidal activity compared to achiral or L‐Glu conjugated nanoparticles, disrupting peptidoglycan synthesis and physically piercing bacterial cell walls, leading to leakage [7].

Aptamers are nucleic acids of unique sequences known for their ability to selectively bind specific targets, including small molecules, proteins, and cells [8]. They have been routinely evolved and selected through a SELEX process [9, 10, 11]. Due to their superior stability and cost‐effectiveness compared to antibodies, aptamers have gained considerable attention in the research fields of biosensing [12, 13, 14, 15, 16, 17, 18] and targeted drug delivery [19, 20, 21, 22] as a binder type. To obtain the aptamers with enhanced target‐binding affinity and selectivity, current approaches are primarily focused on optimizing existing aptamer sequences or developing new selection methods (called “0‐dimensional” approach) [23]. However, there are limited studies of the biological insights and applications of spatially organized aptamers in 3D space. For example, to our best knowledge, no prior studies have investigated the influence of different spatially organized aptamers in 3D space on targeted cell interaction and drug delivery, representing an important gap in the current understanding and utilization of nanostructured aptamer self‐assembly in bioanalytical and bioengineering applications.

Cell surface proteins (CSPs) or biomarkers play many important cellular functions, such as mediating specific cell‐cell and cell‐environment interactions, guiding targeted drug delivery, translating outside cell signals when the CSPs interact with their binders into responses inside cells, triggering an immune response, which may lead to notable and impactful biological and medical outcomes [24, 25]. Herein, we hypothesize that CSPs may have different responses to their nanostructured binders (aptamers used herein) assembled in 3D space with opposite overall chirality and thus, resulting in differential efficacies of cell interactions, cellular internalizations, and cancer drug delivery and cancer cell killing. To test the hypothesis in our study, DNA origami nanostructure (DON) based platform [26] was chosen to build chiral aptamer pattern (or called “CAP”) constructs given that DONs can serve as biocompatible and biostable molecular pegboards with a tunable immunogenicity in 2D or 3D space, with precise structural addressability to arrange external binders, such as aptamers, peptides, proteins, nanoparticles, into patterns with desired spacing and valency at the nanometer scale [27, 28, 29, 30, 31, 32, 33, 34, 35, 36, 37, 38, 39]. They can be affordably produced in quantitative yields [40, 41, 42, 43, 44]. They have shown excellent stability in buffers, cell media, cell lysates, and blood [14, 27, 28, 29, 30, 31, 32, 33, 34, 35], which can be enhanced or finely tuned via surface coating [30, 45, 46, 47], UV‐crosslinking [35], or self‐assembly in aqueous ionic liquid solution [48]. They can be stored for days/months in buffer solutions [14, 49] or as dried materials [43]. All these properties promote the use of DON‐based platform in our study to create CAP constructs focused on understanding CAP structure‐function relationship for achieving better efficacy of targeted cellular interaction, cellular internalization, and ultimately drug delivery.

**FIGURE 1:**
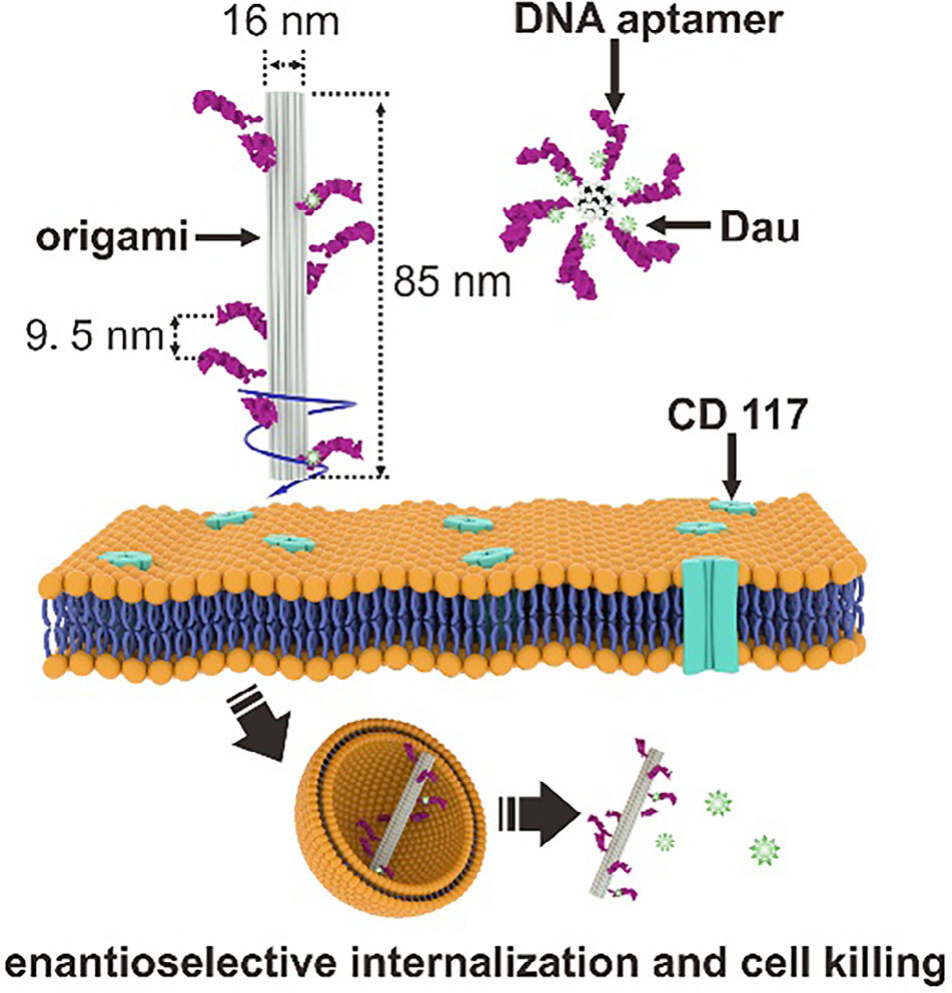
Schematic of DNA origami tube‐templated formation of chiral aptamer pattern (CAP) constructs and realization of CAP‐driven enantioselectivity for acute myeloid leukemia (AML) cell internalization and killing. Tubular‐shaped DNA origami nanostructure (called “DNA tube”) was designed and synthesized to template the spatial arrangement of CD117 protein‐targeting aptamers into either L‐ or R‐form CAP constructs. DNA tube‐aptamers assembly is via DNA hybridization between the docking sites on the DNA tube with the complementary linker sequence carried by the aptamer. Our experiments reveal that CAP constructs exhibit enantioselectivity of cellular interaction, internalization, and cell killing when carrying an anticancer drug, suggesting a promising potential in improved cancer therapeutics.

Specifically, we utilize a tubular‐shaped DON (called “DNA tube” herein) as a molecular pegboard to template the spatial arrangement of CD117‐targeting aptamers [22] into either L‐ or R‐form (Figure 1). We carried out confocal microscopic imaging, flow cytometry, and cell cytotoxicity assays and experiments to test the interactions and cancer drug delivery efficacies offered by L‐ and R‐CAP constructs using CD117 expression cancer cells (i.e., acute myeloid leukemia (AML) cells). Our study revealed that L‐handed CAP built on the DNA tube scaffold demonstrates significantly enhanced cellular internalization efficiency compared to R‐handed CAP built on the same DNA tube scaffold with a different aptamer spatial arrangement using a different set of docking strands. Kinetic studies showed that both L‐CAP and R‐CAP constructs have comparable cell surface attachment rates within the first hour of CAP‐cell incubation. However, an enantioselective uptake process has been observed toward the end of two‐hour CAP‐cell interaction, favoring L‐CAP constructs’ uptake by the cells. Built on these findings, a significant anti‐cancer toxicity for AML cells was observed when using Daunorubicin (Dau)‐loaded L‐CAP construct as a targeted drug delivery vehicle, while R‐CAP construct enabled drug delivery system, only showed minimal cell killing or therapeutic effectiveness.

## 2 Results and Discussion

### 2.1 Design, Synthesis, and Characterization of Tubular‐Shaped DNA Origami Nanostructure and the Templated Formation of L‐ and R‐Handed CAP Constructs

**FIGURE 2:**
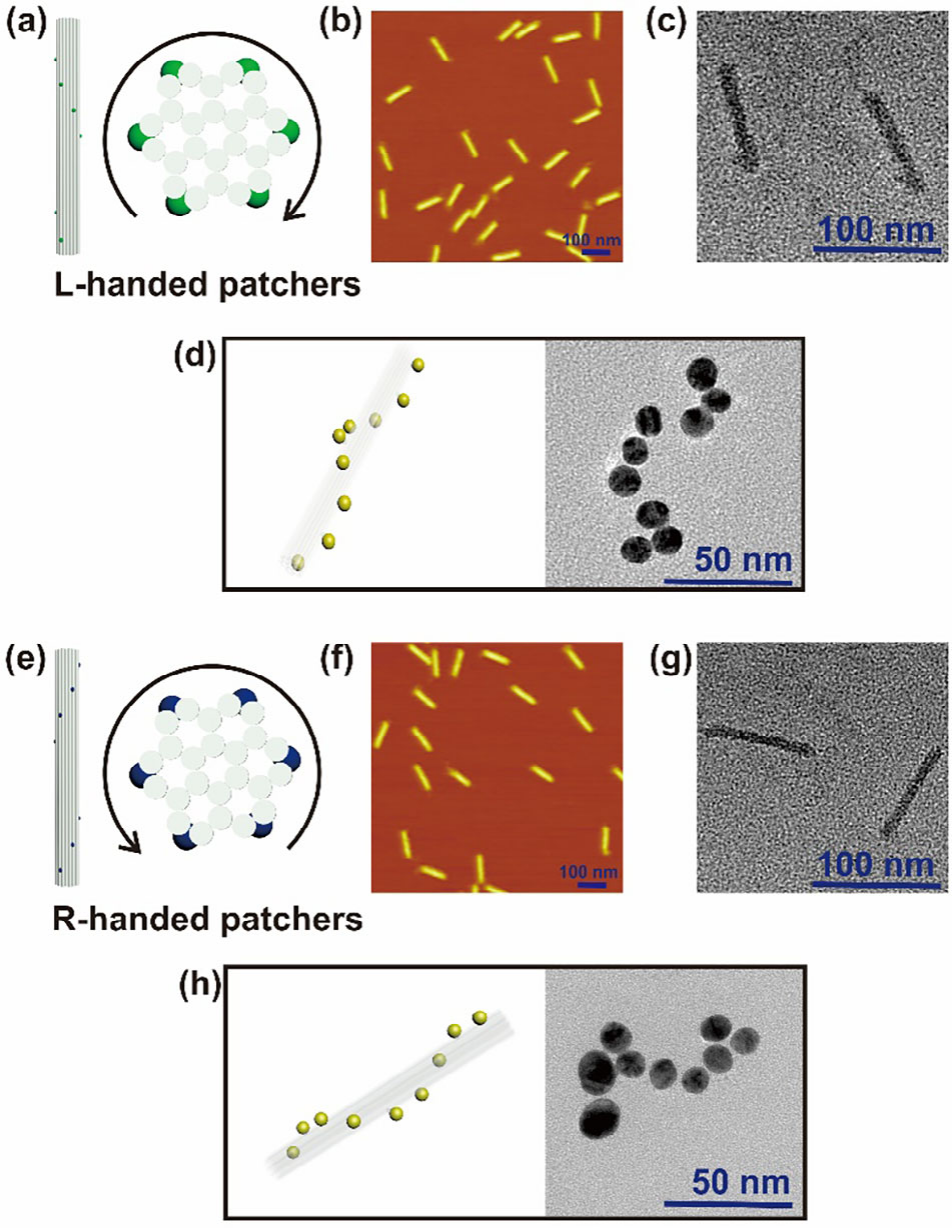
L‐ and R‐handed DNA tube‐aptamers or DNA tube‐AuNPs formation and characterization. (a) Schematic of the side and top views of the DNA tube used to form L‐handed aptamers (CAP) or AuNPs construct. (b) AFM and (c) TEM imaging of the DNA tube used to form L‐handed aptamers (CAP) or AuNPs construct. (d) Schematic and TEM imaging of DNA tube templated L‐handed, CAP‐equivalent assembly of 13‐nm AuNPs. (e) Schematic of the side and top views of the DNA tube used to form R‐handed aptamers (CAP) or AuNPs construct. (f) AFM and (g) TEM imaging of the DNA tube used to form R‐handed aptamers (CAP) or AuNPs construct. (h) Schematic and TEM imaging of DNA tube templated R‐handed, CAP‐equivalent assembly of 13‐nm AuNPs.

We utilized a 24‐helix bundle DNA origami tube (called “DNA tube” herein) [50] that is 101 × 16 nm in size and rigid enough to carry respective single‐stranded DNA (ssDNA) docking sites on the DNA tube surface to arrange external elements (like aptamers and gold nanoparticles (AuNPs) used in our study) into an L‐ or R‐handed construct (Figure 2a,e). The distance between two adjacent docking sites along *Z*‐axis of the DNA tube is estimated to be 9.5 nm by design based on the physical property of dsDNA structure. Tables S1 and S2 list the staple DNA sequences used to assemble the DNA tube scaffold structures for L‐ and R‐handed constructs, respectively. Formation of the DNA tubes (scaffolds for respective assembly of L‐ and R‐CAP constructs) was thoroughly characterized and confirmed by agarose gel electrophoresis (AGE, Figure S1), atomic force microscopy (AFM, Figure 2b,f), and transmission electron microscopy imaging (TEM, Figure 2c,g). Furthermore, we used oxDNA simulation to verify the feasibility of spatially patterning aptamers onto the docking regions on the DNA tube surface, forming CAP constructs via DNA hybridization (Figures S2 and S3). It is worth noting that it is a technical challenge to visualize aptamers using either AFM or TEM imaging to confirm that the designed docking sites on the surface of DNA tubes can precisely arrange the DNA aptamers into an L‐ or R‐handed CAP construct.

To experimentally verify the spatial arrangements of the designed docking sites by addressing such technical challenge, we chose to tag the designed docking site‐complementary DNA sequence with 13 nm AuNPs to form L‐ or R‐handed AuNPs‐DNA tube complexes (Figure S4), in which AuNPs serve as a topological marker under TEM imaging to confirm the chiral configurations of the designed ssDNA docking sites utilized to assemble L‐ or R‐handed CAP constructs (Figure 2d,h). For additional verification of the L‐ or R‐handed conformations, we performed Stochastic Optical Reconstruction Microscopy (STORM) on the L‐ and R‐CAP tubes with Cy5‐labeled small ssDNA linkers attached to the docking sites. The (*x*,*y*) coordinate locations of these Cy5 molecules were identified using Gaussian fitting, and these localizations were overlaid to reconstruct a skeleton of the L and R‐helical conformations of the two designs with sub‐diffraction‐limited resolution (Figure S5a,b). We observe distinct left and right‐handedness for each DNA tube type as evident from the Cy5 signals (Figure S5c–j). The precision of the STORM assay was determined to be ∼7 nm based on overlaying localizations from ∼500 isolated blinking Cy5 molecules (Figure S6).

### 2.2 Enantioselectivity of L‐ and R‐Handed CAP Constructs in Target Cellular Interaction and Internalization

**FIGURE 3:**
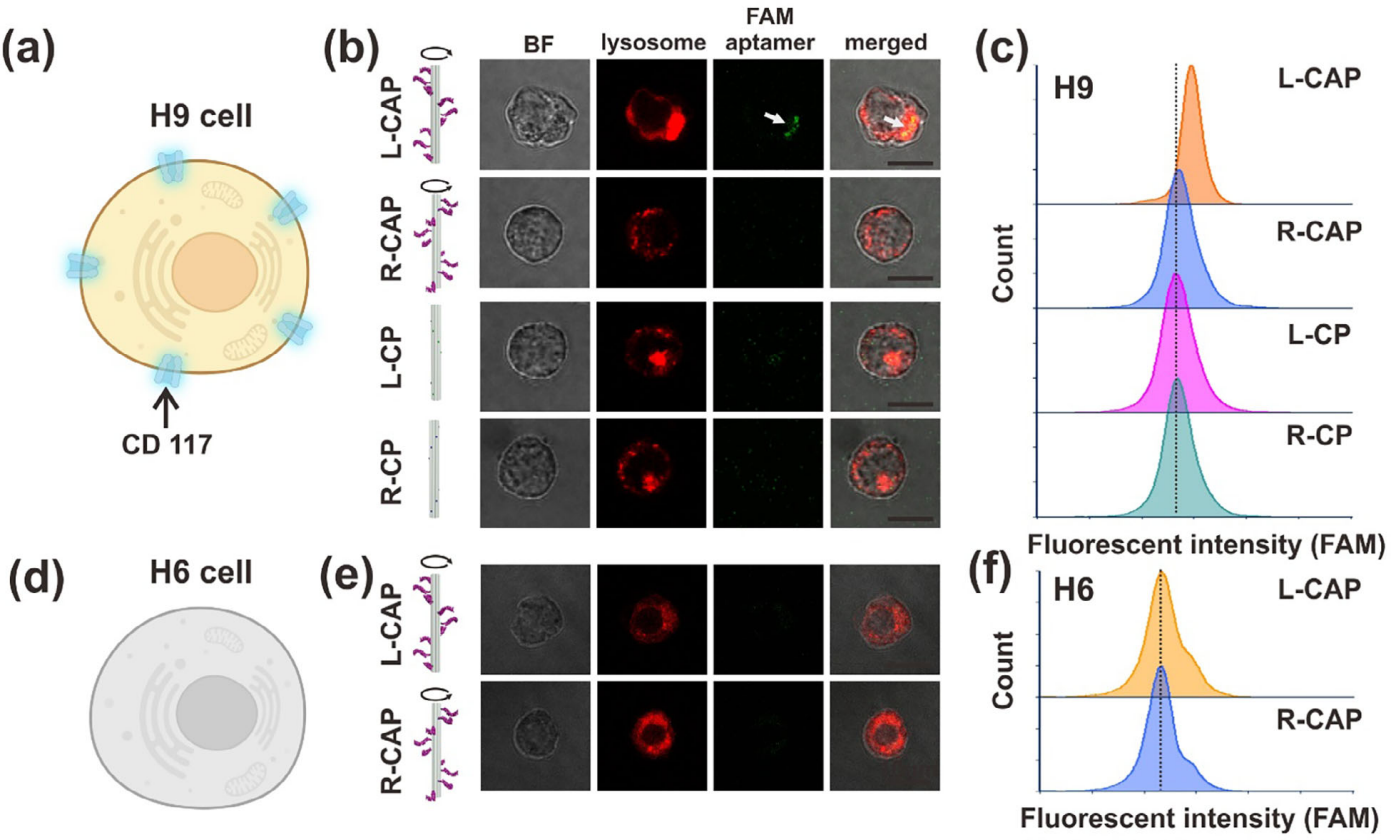
Enantioselectivity of L‐ and R‐handed CAP constructs in target cell internalization. (a) Schematic of an H9 cell that carry CD117 protein biomarkers on the cell outer surface. (b) Confocal microscopy imaging of H9 cells after incubation respectively with L‐handed CAP construct (top row), R‐handed CAP construct (second row), DNA tube (without aptamers) control carrying L‐handed docking sites pattern (third row), and DNA tube (without aptamers) control carrying R‐handed docking sites pattern (fourth row). (c) Comparative flow cytometry analysis of H9 cell interaction respectively with L‐handed CAP, R‐handed CAP, and two DNA tube controls (without aptamers) showing that only L‐handed CAP construct induces a fluorescence signal peak shift, confirming a positive cellular internalization and proving the enantioselectivity of L‐ and R‐handed CAP constructs in target cell internalization. (d) Schematic of an H6 cell whose outer surface does not express any CD117 protein biomarkers. (e) Confocal microscopy imaging of H6 cells after incubation with L‐handed (top row) and R‐handed CAP constructs (bottom row), respectively. No green fluorescence signal was observed with either L‐ or R‐handed CAP construct‐incubated H6 cells, suggesting that the CAP construct‐H9 cell interaction observed by confocal (b) and flow cytometry (c) assays is specifically mediated by CD117‐aptamer binding. The scale bars in (b) and (e) indicate 10 µm. (f) Comparative flow cytometry assays of H6 cell interaction, respectively, with L‐ and R‐handed CAP constructs show no fluorescence signal peak shift. Each confocal imaging or flow cytometry assay is repeated for three times with similar results.

To determine whether the assembled CAP constructs could yield different biological functions, we patterned CD117 protein‐binding aptamers on the DNA tube surface into an L‐ or R‐handed CAP construct. CD117 is a receptor tyrosine kinase and a well‐known cell surface biomarker in various cancers such as acute myeloid leukemia (AML) [51, 52], with prominent roles in the cancer progression. The CD117‐binding aptamer was previously developed by us using in vitro selection, which shows a strong binding to CD117 protein with an equilibrium dissociation constant (*K_D_*) of 23.6 nm (Figure S6). To test the enantioselectivity of L‐ and R‐handed CAP constructs in target cellular interaction and internalization, we incubate CD117 expressing cell, HEL9.1.7(H9) cells, as illustrated in Figure 3a, respectively with L‐ and R‐handed CAP constructs. CD117‐binding aptamer used for making CAP constructs is FAM‐labeled to show a green fluorescence signal under confocal microscopy and flow cytometry for downstream imaging analysis and CAP‐driven cellular interaction quantifications. DNA tubes that only carry L‐ and R‐handed docking sites (without aptamers‐ L/R‐CP, CP being Chiral Particle) are used as negative controls in both assays. Confocal imaging assay confirmed that L‐handed CAP construct can be effectively internalized into the H9 cell after a 2 h incubation, as evidenced by the spatial overlap between the green fluorescence emitted from FAM‐CAP construct with the red fluorescence from the cellular lysosomes after staining with Lysotracker Red (Figure 3b, top row). On the contrary, R‐handed CAP construct shows no green fluorescence after a 2‐h incubation with H9 cells following the same sample processing and confocal imaging assay (Figure 3b, second row). Incubating FAM‐DNA tubes (L/R‐CP)‐based controls with H9 cells for 2 h does not lead to any green fluorescence signal under confocal, confirming the CAP‐cell interaction and internalization are mediated by CD117‐binding aptamers organized on the DNA tube template (Figure 3b, two bottom rows). Such an enantioselectivity of L‐ and R‐handed CAP constructs was further confirmed by the flow cytometry assays, in which an evident shift in the histogram peak for the green fluorescence emitted from the FAM‐aptamer assembled CAP constructs, was observed for the L‐handed CAP construct, but not for the R‐handed CAP construct or DNA tube (L/R‐CP)‐based controls (Figure 3c). To further exclude the possibility that L‐handed CAP construct interacts with non‐targeting (CD117) protein biomarker(s) on H9 cell outer surface, we incubated L‐ and R‐handed CAP constructs respectively with HL‐60 (H6) cells that do not express CD117 proteins (Figure 3d). The confocal images display no obvious H6 cell internalization of CAP constructs (Figure 3e), which aligns with the flow cytometry results that evidenced no detectable shift of the green fluorescence signal emitted from the L‐ or R‐handed CAP construct (Figure 3f).

In summary, these results support our hypothesis that CAP constructs of different chirality could yield different enantioselectivities in target cellular interaction and internalization, with a preference for the CAP construct of L‐handed conformation. Additionally, the L‐handed CAP construct demonstrates an enhanced efficiency of cellular internalization compared to the monomeric aptamer control that barely shows any cellular internalization after a 2 h incubation with the CD117‐expressing H9 cells (Figures S8 and S9).

**FIGURE 4:**
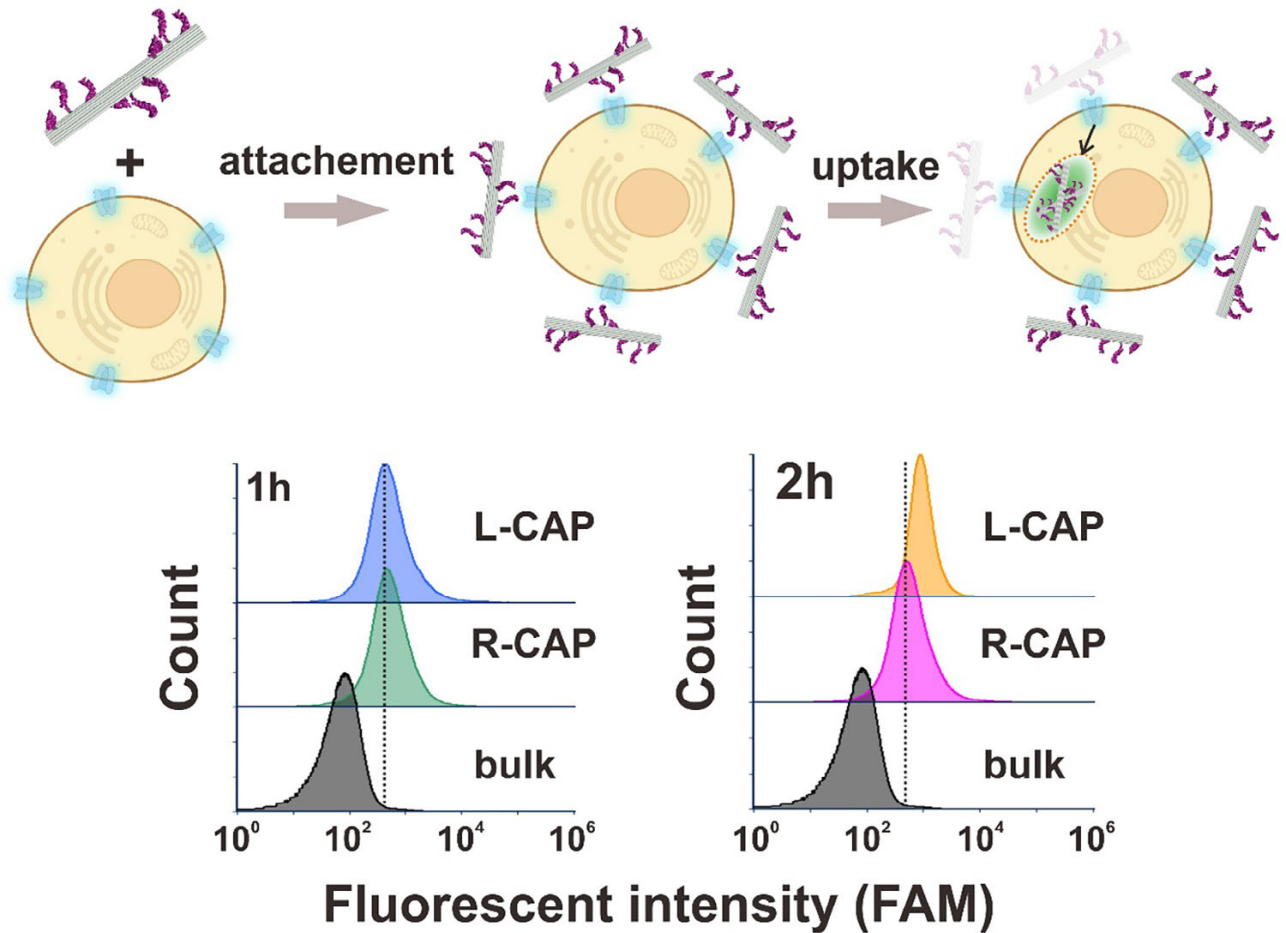
Cellular interaction and internalization of L‐ and R‐handed CAP constructs at different incubation time points. Top row: A schematic drawing of cellular interaction and internalization of CAP constructs. Bottom row: Flow cytometry data reveal that L‐ and R‐handed CAP constructs show similar fluorescence signal peak shift after a 1 h incubation with the target cells, indicating a similar degree of cell surface CD117 protein binding between two CAP constructs; However, a more significant fluorescence signal shift for L‐handed CAP construct has been observed post a 2 h incubation with the target cells, suggesting that L‐handed CAP construct could induce a better target cell internalization after binding with the CD117 proteins on the cell outer surface. Each flow cytometry experiment is repeated for three times with similar results.

The above confocal imaging and flow cytometry assays have shown the CAP constructs of different chirality can induce distinct target cell internalization effects after a 2 h incubation (Figure 3). To provide a more mechanistic understanding of what has caused such difference, we carried out additional flow cytometry experiments to monitor the degree of CAP construct‐H9 cell interactions with a better temporal resolution by adding a 1 h CAP construct‐H9 cell incubation time point. Interestingly, as shown in Figure 4, the interaction between H9 cells with L‐ or R‐handed CAP construct induces a similar degree of CAP construct‐H9 cell interaction, as indicated by a similar degree of peak shift in flow cytometry assays. However, L‐handed CAP construct‐cell interaction starts to yield a more significant peak shift after a 2 h incubation, indicating a more cellular internalization of the CAP construct. These data suggest that the chiral property of spatially patterned aptamers plays an important role in determining the degree of CAP construct uptake by the target cells, while the aptamer itself, regardless of its spatially chiral patterns, retains the binding ability to the target protein on the cell outer surface.

### 2.3 Potential Mechanism of Enantio‐Selectivity in L‐ and R‐Handed CAP Constructs

**FIGURE 5:**
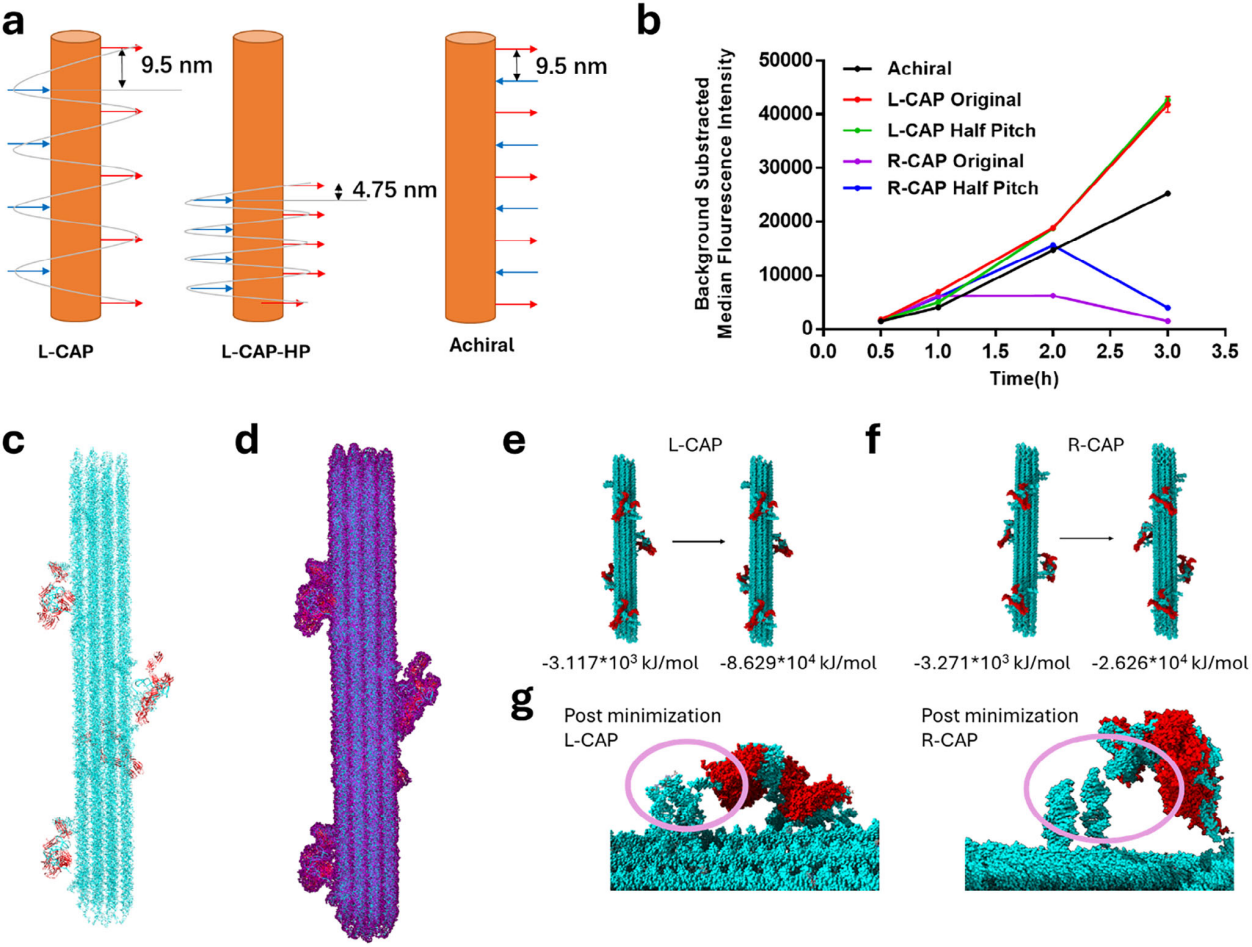
Elucidating the possible mechanism of enantioselectivity of L‐CAP structures. (a) Design strategy of the original L‐CAP designs compared to L‐CAP‐Half Pitch/HP to change inter‐aptamer and distance and Achiral to remove handedness. (b) Flow cytometry‐based comparison of cell binding and/or internalization of all five designs reveal potential of L‐handedness in cellular binding and subsequent internalization. Values represent the mean and standard error of 6 independent replicates. (c) Representative L‐CAP design harboring four dimer complexes, each have two aptamers interacting with a CD117 monomer that form the physiological CD117 dimer. (d) Solvation of the L‐CAP design using the water model shell of 30 Å. (e) Pre‐ and post‐minimization energies of the L‐CAP design. (f) Pre‐ and post‐minimization energies of the R‐CAP design. (g) Close up view of a dimer complex attached to the CAP structure reveals detachment of one aptamer from the CAP dock site in R‐design. The L‐design however remain intact highlighting the enantioselectivity mediated aptamer conformation resulting in selective binding and internalization.

Our experimental findings suggest that the L‐handed CAP construct promotes cellular internalization by inducing dimerization of CD117 proteins (CD117 dimer model shown in Figure S10) on the cell surface. This observation aligns with prior studies showing that CD117 dimerizes through its extracellular domains upon binding to exogenous partners, a process that triggers downstream signaling and internalization [53]. In contrast, the R‐handed CAP construct seems to bind in a way that prevents CD117 dimerization, thereby limiting effective internalization. These differences likely arise from either the structural handedness of the CAP constructs, the spacing between aptamers, or a combination of both. Each CAP design carries nine aptamers in either L‐ or R‐handed orientations, with 9‐nm vertical spacing. To test whether aptamer distance affects internalization, additional constructs were designed: L‐CAP‐HP and R‐CAP‐HP (HP is half‐pitch, 4.5‐nm vertical spacing between aptamers), and an achiral version with aptamers in a straight line at 9‐nm vertical intervals (Figures 5a; S11). H9 cells were incubated with these constructs, and flow cytometry measured binding/internalization over time. All five constructs (L‐CAP, L‐CAP‐HP, R‐CAP, R‐CAP‐HP, achiral) bound to cells within 0.5 h. L‐CAP and L‐CAP‐HP showed steadily increasing signals, indicating continuous uptake up to 3 h (Figures 5b; S12a–e). In contrast, R‐CAP displayed stable signals at 1–2 h but declined by 3 h, suggesting weak or nonspecific binding and subsequent detachment. R‐CAP‐HP showed a delayed increase followed by a rapid decline (Figures 5b; S12a–e). Interestingly, the achiral construct behaved like L‐CAP with an increase in signal up to 3 h, albeit at lower intensity, indicating that handedness, not just spacing, governs internalization (Figures 5b; S12a–e). It also suggests that the local concentration of the aptamers on the CAP also does not govern internalization. Control experiments confirmed that CAP constructs remained structurally intact after 3 h of incubation with Ramos cells (does not express CD117) (Figure S12f). Overall, these results highlight that only L‐handed CAP enables effective CD117 dimerization and subsequent internalization.

To further investigate the thermodynamics and mechanism behind CAP handedness, we carried out ab initio structural modeling of both L‐ and R‐handed CAP constructs with aptamers attached to their target protein, CD117. While computational modeling of large DNA nanostructures such as DNA origami has been performed before [54, 55], no previous studies have extended this approach to DNA frameworks decorated with protein moieties. This gap led us to develop a specialized protocol for modeling CAP constructs bound to CD117. Because molecular dynamics simulations are computationally intensive for systems of this size, we chose to focus on energy minimization instead. Our process began with docking the experimentally determined monomer of CD117 to an aptamer generated by ab initio modeling. The docked aptamer‐protein complex was then energy minimized and paired to form a dimeric assembly, mimicking CD117 natural dimerization state (Figure S13). One challenge was that the available CD117 structure lacked the transmembrane domain as well as the cell membrane acting as an anchor, which increases the risk of proteins drifting away from CAP‐aptamer sites during subsequent minimization. To address this, we introduced covalent C–C bonds between aptamer nucleotides and specific residues on CD117 (Val214, Trp261, Phe267, Tyr291, Phe296) using PyMOL editing. Next, four copies of the dimer complex were attached to both L‐ and R‐CAP models using oxDNA's pair selection utility (Figure S14). The CD117 dimers were oriented on the CAP so their domain V regions pointed outward—consistent with the protein's natural membrane anchoring. Each CAP structure, therefore, contained four CD117 dimers correctly aligned and attached through aptamers (representative L‐CAP shown in Figure 5c). The complete models were solvated using the TIP3P water model, counter‐ions added using the AMBER forcefield, and then minimized over 5000 steps (Figure 5d).

The purpose of this minimization was to determine whether CD117 dimers would remain intact or dissociate depending on CAP handedness. Both L‐ and R‐CAP models showed overall decrease in free energy, but the L‐CAP exhibited significantly lower final energy, indicating a more stable system (Figure 5e,f). Closer inspection revealed that while aptamers in the L‐CAP remained firmly attached to dock sites, the R‐CAP displayed detachment of few aptamers from its dock sites around the 3000th minimization step (Figures 5g; S15). This detachment occurred after the steepest descent phase, suggesting that the rigidity of the R‐CAP makes it incompatible with stable accommodation of CD117 dimers, despite inherent flexibility of both aptamers and dock sites. Similar structural denaturation of DNA under computation minimization has been noted in prior studies [56, 57, 58, 59]. To confirm whether this detachment was simply an artifact of the covalent linkages introduced, we evaluated the thermodynamic feasibility of the observed separation. The six covalent bonds between aptamers and proteins contributed approximately ∼1700 kJ/mol of stability. In contrast, dissociating 15 G–C dock site ligations would theoretically require ∼1650 kJ/mol. Additionally, CD117 dimerization itself contributes a large negative free energy (∼ −4.9 × 10^3^ kJ/mol), strongly favoring dimer stability. Together, these calculations suggest that the R‐CAP configuration is inherently less favorable thus forcing CD117 dimers’ geometry to destabilize the aptamer‐dock interactions, ultimately leading to aptamer detachment. In summary, our modeling demonstrates that CAP handedness has a direct thermodynamic impact, where only L‐CAP stabilizes CD117 dimers and maintains aptamer docking.

Taken together, our experimental and computational data suggest that while the R‐handed CAP construct allows binding of the individual aptamers to CD117, such a conformation does not lead to dimerization and subsequent internalization. As the formation of the dimer is critical toward a CAP construct internalization, R‐CAP design fails to enter the cells and eventually dissociates from the proteins on the cell surface.

### 2.4 Anti‐Cancer Effects of Drug‐Loaded CAP Constructs

**FIGURE 6:**
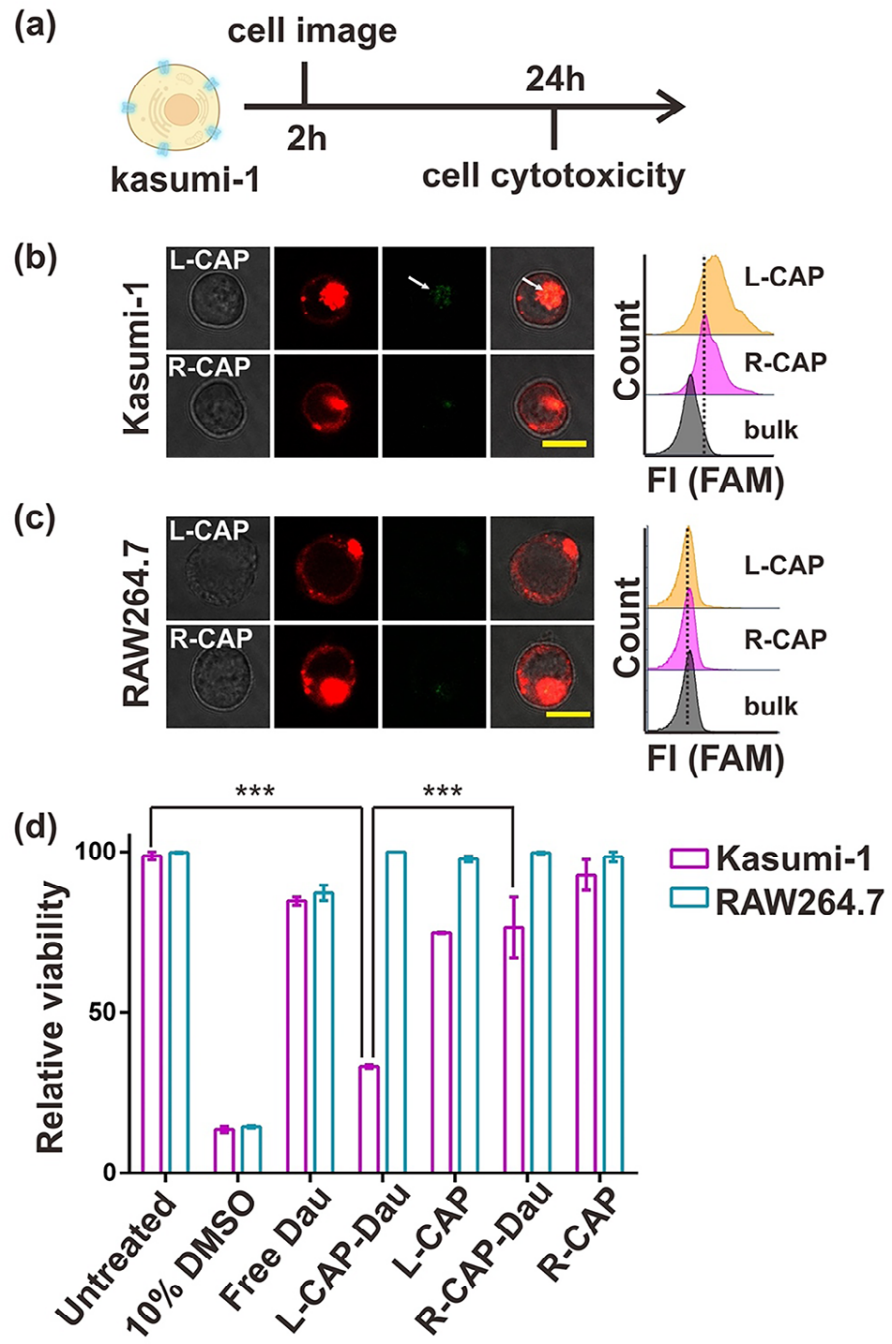
Acute myeloid leukemia (AML) cancer cell imaging and cytotoxicity assay post treatment with daunorubicin (Dau) loaded CAP constructs. (a) Schematic of the time points for cell imaging and cell cytotoxicity assays. (b) Confocal imaging (left) and flow cytometry (right) assays show the level of K‐1 cell uptake of Dau‐loaded L‐handed CAP construct is higher than the Dau‐loaded R‐handed CAP construct. (c) Confocal imaging (left) and flow cytometry (right). Assays using RAW264.7 cells as a non‐CD117 expressing cell control show the level of RAW264.7 cell uptake of Dau‐loaded L‐ and R‐handed CAP constructs is highly similar with each other and same as the background signal. Each confocal or flow cytometry experiment is repeated for three times with similar results. The scale bars in (b) and (c) indicate 10 µm. (d) Cell viability or cytotoxicity assays confirm an enantioselectivity of Dau‐loaded L‐ and R‐handed CAP constructs in cancer cell killing. Data per condition represents mean and standard error for 6 independent replicates. Significance is indicated by p values (^***^<0.005) and determined by unpaired two‐tailed Student's *t*‐test.

Selective binding of the aptamers to target proteins upon arrangement in an L‐handed chiral conformation opens a wider avenue of developing designer DNA nanostructure (DDN)‐based vehicles for multiple applications including targeted drug delivery. Chiral switches have been previously shown to be utilized in clinical applications [60, 61]. As mentioned earlier, CD117 is a key cancer biomarker in AML and is utilized for various diagnostic and therapeutic discoveries. Thus, we aim to determine the effect of different CAP constructs on anti‐cancer outcomes (Figure 6a). To explore the internalization of aptamers with different chiral patterns into K1 and RAW264.7 cells, cell images were taken 2 h post‐incubation as a previously justified timepoint. Strong internalization, as indicated by the green fluorescence signal, emitted by the CAP constructs, on confocal images and flow cytometry results, was only observed with the L‐handed CAP construct (Figures 6b,c; S12), which is consistent with the above results shown in Figures 3 and 4. Subsequently, the CD117‐binding aptamer was first loaded with daunorubicin (Dau), a well‐characterized anti‐tumor drug used in AML clinical settings [62, 63]. The Dau‐loaded aptamers were then arranged on the respective DNA tube scaffolds to make L‐ and R‐handed CAP‐Dau constructs for treating K‐1 (target) and Raw (control) cells for 24 h. Dau shows minimal cytotoxic effects in free state. Similarly, when arranged in an R‐handed conformation, the Dau‐loaded CAP construct‐enabled treatment shows a mild reduction in viability of K‐1 cells. However, we observed a significant reduction in viability upon K‐1 cells treatment with Dau‐loaded aptamers arranged specifically in the L‐handed conformation (Figure 6d).

CD117 (also known as c‐Kit) is a protooncogene and plays key functions in the progression of various cancers such as AML, melanoma and stromal tumors [64]. Concurrently, CD117 has also been leveraged as a target for the development of anti‐cancer therapeutics. CD117 monomers are comprised of immunoglobulin‐like domains (Figure S16). The dimerization of CD117 is critical for the effective internalization and induction of cell death. While the R‐handed CAP construct does exhibit binding with the cell outer surface CD117, the binding conformation fails to induce dimerization, causing CD117 to remain on the cell outer membrane. However, the L‐handed CAP construct not only binds to CD117 but does so in a conformation that allows dimerization of CD117. This dimerization triggers subsequent internalization of the complex that facilitates the delivery of Dau drug into the cells. The internalized Dau causes DNA damage that results in cell death. In summary, these results suggest that the chiral arrangement of the aptamers not only affects the internalization of a CAP construct into the target cells but can also be leveraged to selectively deliver small molecules, such as anti‐cancer drugs, for various desired therapeutic outcomes.

## 3 Conclusion

A lot of cancer drugs (small molecules, siRNA, mRNA, peptides, etc.) have been produced or identified with great potential. However, a key roadblock to deploying them to create therapeutic opportunities in oncology is the off‐target effects given the lack of targeted drug delivery. Previous studies have demonstrated that engineered nucleic acid nanostructures, such as DNA origami nanostructures, have great biocompatibility and biostability in vivo with tunable immunogenicity [26], and have been used in therapeutics [65, 66]. The recent studies have demonstrated that designer DNA nanostructure (DDN)‐based platforms have excellent addressability to arrange multiplex molecular binders into desired patterns with nanometer scale accuracy, offering high virus and cell binding strength, increasing affinity by thousands to millions of folds through multivalent and pattern‐matching interactions between the binders on DDNs and proteins on virus or cell outer surfaces [22,14, 15, 18, 67, 68, 69, 70, 71, 72, 73, 74]. The resulting high binding affinity could realize a great potential to promote targeted drug delivery for improved disease treatment efficacies.

Most biomolecules and their assemblies, such as proteins and DNA, share a fundamental geometrical characteristic: chirality, or mirror asymmetry. This property leads to distinct biological, chemical, and physical changes and functions. By targeting CD117 protein, a key biomarker playing critical functions in the progression of various cancers, we have revealed that the L‐handed chiral arrangement of the aptamers not only improve the binding affinity to the target cell via multivalent interactions, but also promote the cellular uptake of the chiral aptamer patterned (CAP) construct, speculated by inducing dynamic CD117 protein dimerization on cell surface. Such a hypothesis aligns very well with the prior studies showing that CD117 can dimerize when its extracellular immunoglobulin‐like domains bind to various exogenous binders, which could initiate the CD117 cellular signaling and the subsequent complex cellular internalization [53]. On the contrary, R‐handed CAP construct may bind to the cell in a way not promoting the formation of dimerized CD117 proteins on the cell surface, thus preventing its effective internalization. The performance of the L‐handed CAP construct for reducing viability of CD117‐expressing cells in vitro shows promising therapeutic potential. We present our hypothesis on the mechanism of this enantioselective binding and internalization by L‐CAP design in Figure S17. A detailed mechanistic hypothesis for how L‐CAP‐induced CD117 dimerization leads to internalization of the resulting complex (L‐CAP with multiple CD117 dimers) is presented in Note S1 and Figure S18. This observation is consistent with a recent study reporting that the left‐handed nanostructures have shown biological relevant functionality. In this study, left‐handed nanostructures exhibited faster rotational dynamics at physiological salt concentrations compared to right‐handed counterparts, an effect attributed to chirality‐dependent anisotropies in hydrodynamic and electrophoretic mobility [75]. Such differences in nanoscale rotational dynamics could influence how chiral nanostructures engage with and reorganize membrane proteins, thereby modulating downstream cellular uptake. As binders specific to other cancer cell surface protein biomarkers can be evolved or synthesized, our strategy could be widely applicable by including a new element‐ L‐handed chiral binder (aptamer and nanobody) pattern in addition to multivalent interactions, towards the design of a DDN‐based targeted drug delivery vehicle for improved disease treatment efficacies.

## Author Contributions

T.S., A.D., and X.W. conceptualized the study. T.S., with the assistance from D.G., M.Z., X.W., and L.Z., designed, synthesized, and characterized the DNA origami and its‐templated aptamer and AuNP constructs using gel electrophoresis. T.S. performed SPR, AFM, and TEM experiments. T.S. and A.D. performed confocal microscopy imaging experiments. A.D. performed flow cytometry and cell toxicity assays. A.D., Y.A., and L.Z. performed computational modelling and simulation assays. G.C. and Y.Z. performed super‐resolution imaging. All authors performed data analysis. T.S., A.D., and X,W. led the preparation of the manuscript. All authors contribute to the review and editing of the manuscript. T.S. and A.D. contributed equally to this work. X.W. supervised the study.

## Conflicts of Interest

The authors declare no conflicts of interest.

## Supporting information




**Supporting File**: adma72153‐sup‐0001‐SuppMat.pdf.

## Data Availability

The data that support the findings of this study are available in the supplementary material of this article.

## References

[1] W. Acevedo and P. A. Temussi , “The Origin of Unpleasant Aftertastes in Synthetic Sweeteners: A Hypothesis,” Frontiers in Molecular Biosciences 5 (2019): 119. 30713843 10.3389/fmolb.2018.00119 PMC6345712

[2] D. M. Eckert , V. N. Malashkevich , L. H. Hong , P. A. Carr , and P. S. Kim , “Inhibiting HIV‐1 Entry,” Cell 99, no. 1 (1999): 103–115, 10.1016/S0092-8674(00)80066-5. 10520998

[3] H.‐E. Lee , H.‐Y. Ahn , J. Mun , et al., “Amino‐Acid‐ and Peptide‐Directed Synthesis of Chiral Plasmonic Gold Nanoparticles,” Nature 556, no. 7701 (2018): 360–365, 10.1038/s41586-018-0034-1. 29670265

[4] J. Cai , C. Hao , M. Sun , W. Ma , C. Xu , and H. Kuang , “Chiral Shell Core–Satellite Nanostructures for Ultrasensitive Detection of Mycotoxin,” Small 14, no. 13 (2018): 1703931, 10.1002/smll.201703931. 29424128

[5] J. Sachs , J.‐P. Günther , A. G. Mark , and P. Fischer , “Chiroptical Spectroscopy of a Freely Diffusing Single Nanoparticle,” Nature communications 11, no. 1 (2020): 4513, 10.1038/s41467-020-18166-5. PMC7481242 32908138

[6] Y. Li , Z. Miao , Z. Shang , Y. Cai , J. Cheng , and X. Xu , “A Visible‐ and NIR‐Light Responsive Photothermal Therapy Agent by Chirality‐Dependent MoO_3−X_ Nanoparticles,” Advanced Functional Materials 30, no. 4 (2019): 1906311.

[7] M. Zhang , H. Zhang , J. Feng , Y. Zhou , and B. Wang , “Synergistic Chemotherapy, Physiotherapy and Photothermal Therapy Against Bacterial and Biofilms Infections Through Construction of Chiral Glutamic Acid Functionalized Gold Nanobipyramids,” Chemical Engineering Journal 393 (2020): 124778.

[8] D. Chang , Z. Wang , C. D. Flynn , et al., “A High‐Dimensional Microfluidic Approach for Selection of Aptamers With Programmable Binding Affinities,” Nature Chemistry 15, no. 6 (2023): 773–780, 10.1038/s41557-023-01207-z. 37277648

[9] A. Brown , J. Brill , R. Amini , C. Nurmi , and Y. Li , “Development of Better Aptamers: Structured Library Approaches, Selection Methods, and Chemical Modifications,” Angewandte Chemie International Edition 63, no. 16 (2024): 202318665, 10.1002/anie.202318665. 38253971

[10] Y. Ding and J. Liu , “Pushing Adenosine and ATP SELEX for DNA Aptamers With Nanomolar Affinity,” Journal of the American Chemical Society 145, no. 13 (2023): 7540–7547, 10.1021/jacs.3c00848. 36947745

[11] M. C. DeRosa , A. Lin , P. Mallikaratchy , et al., “In Vitro Selection of Aptamers and Their Applications,” Nature Reviews Methods Primers 3, no. 1 (2023): 54, 10.1038/s43586-023-00238-7. PMC10647184 37969927

[12] Q. Mou , X. Xue , Y. Ma , et al., “Efficient Delivery of a DNA Aptamer‐Based Biosensor Into Plant Cells for Glucose Sensing Through Thiol‐Mediated Uptake,” Science Advances 8, no. 26 (2022): abo0902, 10.1126/sciadv.abo0902. PMC9242441 35767607

[13] C. Ye , M. Wang , J. Min , et al., “A Wearable Aptamer Nanobiosensor for Non‐Invasive Female Hormone Monitoring,” Nature Nanotechnology 19, no. 3 (2024): 330–337, 10.1038/s41565-023-01513-0. PMC10954395 37770648

[14] P. S. Kwon , S. Ren , S. J. Kwon , et al., “Designer DNA Architecture Offers Precise and Multivalent Spatial Pattern‐Recognition for Viral Sensing and Inhibition,” Nature Chemistry 12, no. 1 (2020): 26–35, 10.1038/s41557-019-0369-8. PMC6925649 31767992

[15] N. Chauhan , Y. Xiong , S. Ren , et al., “Net‐Shaped DNA Nanostructures Designed for Rapid/Sensitive Detection and Potential Inhibition of the SARS‐CoV‐2 Virus,” Journal of the American Chemical Society 145, no. 37 (2023): 20214–20228, 10.1021/jacs.2c04835. 35881910 PMC9344894

[16] H. Lee , W. Wang , N. Chauhan , et al., “Rapid Detection of Intact SARS‐CoV‐2 Using Designer DNA Nets and a Pocket‐Size Smartphone‐Linked Fluorimeter,” Biosensors and Bioelectronics 229 (2023): 115228, 10.1016/j.bios.2023.115228. 36963325 PMC10019040

[17] S. Umrao , M. Zheng , X. Jin , S. Yao , and X. Wang , “Net‐Shaped DNA Nanostructure‐Based Lateral Flow Assays for Rapid and Sensitive SARS‐CoV‐2 Detection,” Analytical Chemistry 96, no. 8 (2024): 3291–3299, 10.1021/acs.analchem.3c03698. 38306661 PMC10922791

[18] L. Zhou , Y. Xiong , A. Dwivedy , et al., “Bioinspired Designer DNA NanoGripper for Virus Sensing and Potential Inhibition,” Science Robotics 9, no. 96 (2024): adi2084, 10.1126/scirobotics.adi2084. PMC11750070 39602515

[19] Z. Huang , D. Wang , Q. Zhang , Y. Zhang , R. Peng , and W. Tan , “Leveraging Aptamer‐Based DNA Nanotechnology for Bioanalysis and Cancer Therapeutics,” Accounts of Materials Research 5, no. 4 (2024): 438–452, 10.1021/accountsmr.3c00249.

[20] A. D. Keefe , S. Pai , and A. Ellington , “Aptamers as Therapeutics,” Nature Reviews Drug Discovery 9, no. 7 (2010): 537–550, 10.1038/nrd3141. 20592747 PMC7097324

[21] L. Kelly , K. E. Maier , A. Yan , and M. Levy , “A Comparative Analysis of Cell Surface Targeting Aptamers,” Nature Communications 12, no. 1 (2021): 6275, 10.1038/s41467-021-26463-w. PMC8560833 34725326

[22] A. Dwivedy , D. Baskaran , G. Sharma , et al., “Engineering Novel DNA Nanoarchitectures for Targeted Drug Delivery and Aptamer Mediated Apoptosis in Cancer Therapeutics,” Advanced Functional Materials 35, no. 22 (2025): 2425394, 10.1002/adfm.202425394. 40487293 PMC12143806

[23] M. C. DeRosa , A. Lin , P. Mallikaratchy , et al., “In Vitro Selection of Aptamers and Their Applications,” Nature Reviews Methods Primers 3, no. 1 (2023): 54, 10.1038/s43586-023-00238-7. PMC10647184 37969927

[24] X. Ye , J. A. Kaczmarczyk , and B. Luke , “Cell Surface Protein Enrichment for Biomarker and Drug Target Discovery Using Mass Spectrometry‐based Proteomics,” in Proteomic and Metabolomic Approaches to Biomarker Discovery (Academic Press, 2020), 409–420.

[25] N. Abazova and J. Krijgsveld , “Advances in Stem Cell Proteomics,” Current Opinion in Genetics & Development 46 (2017): 149–155, 10.1016/j.gde.2017.07.007. 28806595

[26] P. Zhan , A. Peil , Q. Jiang , et al., “Recent Advances in DNA Origami‐Engineered Nanomaterials and Applications,” Chemical Reviews 123, no. 7 (2023): 3976–4050, 10.1021/acs.chemrev.3c00028. 36990451 PMC10103138

[27] Q. A. Mei , X. X. Wei , F. Y. Su , et al., “Stability of DNA Origami Nanoarrays in Cell Lysate,” Nano Letters 11, no. 4 (2011): 1477–1482, 10.1021/nl1040836. 21366226 PMC3319871

[28] J. Hahn , S. F. J. Wickham , W. M. Shih , and S. D. Perrault , “Addressing the Instability of DNA Nanostructures in Tissue Culture,” ACS Nano 8, no. 9 (2014): 8765–8775, 10.1021/nn503513p. 25136758 PMC4174095

[29] N. P. Agarwal , M. Matthies , F. N. Gur , K. Osada , and T. L. Schmidt , “Block Copolymer Micellization as a Protection Strategy for DNA Origami,” Angewandte Chemie International Edition 56, no. 20 (2017): 5460–5464, 10.1002/anie.201608873. 28295864

[30] S. D. Perrault and W. M. Shih , “Virus‐Inspired Membrane Encapsulation of DNA Nanostructures To Achieve in Vivo Stability,” Acs Nano 8, no. 5 (2014): 5132–5140, 10.1021/nn5011914. 24694301 PMC4046785

[31] S. Li , Q. Jiang , S. Liu , et al., “A DNA Nanorobot Functions as a Cancer Therapeutic in Response to a Molecular Trigger in Vivo,” Nature Biotechnology 36, no. 3 (2018): 258–264, 10.1038/nbt.4071. 29431737

[32] M. E. Kizer , Y. Deng , G. Kang , P. E. Mikael , X. Wang , and A. J. Chung , “Hydroporator: A Hydrodynamic Cell Membrane Perforator for High‐Throughput Vector‐Free Nanomaterial Intracellular Delivery and DNA Origami Biostability Evaluation,” Lab on a Chip 19, no. 10 (2019): 1747–1754, 10.1039/C9LC00041K. 30964485

[33] D. Jiang , Z. Ge , H. J. Im , et al., “DNA Origami Nanostructures Can Exhibit Preferential Renal Uptake and Alleviate Acute Kidney Injury,” Nature Biomedical Engineering 2, no. 11 (2018): 865–877, 10.1038/s41551-018-0317-8. PMC6258029 30505626

[34] Y. Kim and P. Yin , “Enhancing Biocompatible Stability of DNA Nanostructures Using Dendritic Oligonucleotides and Brick Motifs,” Angewandte Chemie International Edition 59, no. 2 (2020): 700–703, 10.1002/anie.201911664. 31595637 PMC6940523

[35] T. Gerling , M. Kube , B. Kick , and H. Dietz , “Sequence‐Programmable Covalent Bonding of Designed DNA Assemblies,” Science Advances 4, no. 8 (2018): aau1157, 10.1126/sciadv.aau1157. PMC6097813 30128357

[36] P. W. Rothemund , “Folding DNA to Create Nanoscale Shapes and Patterns,” Nature 440, no. 7082 (2006): 297–302, 10.1038/nature04586. 16541064

[37] A. R. Chandrasekaran , N. Anderson , M. Kizer , K. Halvorsen , and X. Wang , “Beyond the Fold: Emerging Biological Applications of DNA Origami,” Chembiochem 17, no. 12 (2016): 1081–1089, 10.1002/cbic.201600038. 26928725

[38] O. I. Wilner and I. Willner , “Functionalized DNA Nanostructures,” Chemical Reviews 112, no. 4 (2012): 2528–2556, 10.1021/cr200104q. 22233123

[39] X. Luo , D. Saliba , T. Yang , et al., “Minimalist Design of Wireframe DNA Nanotubes: Tunable Geometry, Size, Chirality, and Dynamics,” Angewandte Chemie International Edition 62, no. 44 (2023): 202309869, 10.1002/anie.202309869. 37610293

[40] C. Ducani , C. Kaul , M. Moche , W. M. Shih , and B. Hogberg , “Enzymatic Production of 'Monoclonal Stoichiometric' Single‐Stranded DNA Oligonucleotides,” Nature Methods 10, no. 7 (2013): 647–652, 10.1038/nmeth.2503. 23727986 PMC3843646

[41] S. Kosuri and G. M. Church , “Large‐Scale De Novo DNA Synthesis: Technologies and Applications,” Nature Methods 11, no. 5 (2014): 499–507, 10.1038/nmeth.2918. 24781323 PMC7098426

[42] B. Kick , F. Praetorius , H. Dietz , and D. Weuster‐Botz , “Efficient Production of Single‐Stranded Phage DNA as Scaffolds for DNA Origami,” Nano Letters 15, no. 7 (2015): 4672–4676, 10.1021/acs.nanolett.5b01461. 26028443 PMC4532261

[43] F. Praetorius , B. Kick , K. L. Behler , M. N. Honemann , D. Weuster‐Botz , and H. Dietz , “Biotechnological Mass Production of DNA Origami,” Nature 552, no. 7683 (2017): 84–87, 10.1038/nature24650. 29219963

[44] S. Palluk , D. H. Arlow , T. de Rond , et al., “De Novo DNA Synthesis Using Polymerase‐Nucleotide Conjugates,” Nature Biotechnology 36, no. 7 (2018): 645–650, 10.1038/nbt.4173. 29912208

[45] H. Auvinen , H. Zhang , A. Kopilow , et al., “Protein Coating of DNA Nanostructures for Enhanced Stability and Immunocompatibility,” Advanced Healthcare Materials 6, no. 18 (2017): 1700692, 10.1002/adhm.201700692. 28738444

[46] N. Ponnuswamy , M. M. C. Bastings , B. Nathwani , et al., “Oligolysine‐Based Coating Protects DNA Nanostructures from Low‐Salt Denaturation and Nuclease Degradation,” Nature Communications 8 (2017): 15654, 10.1038/ncomms15654. PMC5460023 28561045

[47] F. M. Anastassacos , Z. Zhao , Y. Zeng , and W. M. Shih , “Glutaraldehyde Cross‐Linking of Oligolysines Coating DNA Origami Greatly Reduces Susceptibility to Nuclease Degradation,” Journal of the American Chemical Society 142, no. 7 (2020): 3311–3315, 10.1021/jacs.9b11698. 32011869

[48] D. Gandavadi , H. Talbot , A. Dwivedy , et al., “DNA Nanostructure Self‐Assembly in an Aqueous Ionic Liquid Solution With Enhanced Stability and Target Binding Affinity,” Journal of the American Chemical Society 147, no. 46 (2025): 42545–42635, 10.1021/jacs.5c13969. PMC12706753 41187335

[49] Y. Deng , M. Kizer , M. Rada , et al., “Intracellular Delivery of Nanomaterials via an Inertial Microfluidic Cell Hydroporator,” Nano Letters 18, no. 4 (2018): 2705–2710, 10.1021/acs.nanolett.8b00704. 29569926

[50] A. Kuzyk , R. Schreiber , Z. Fan , et al., “DNA‐Based Self‐Assembly of Chiral Plasmonic Nanostructures With Tailored Optical Response,” Nature 483, no. 7389 (2012): 311–314, 10.1038/nature10889. 22422265

[51] F. Zhao , Y. Chen , Q. Wu , Z. Wang , and J. Lu , “Prognostic Value of CD117 in Cancer: A Meta‐Analysis,” International Journal of Clinical and Experimental Pathology 7, no. 3 (2014): 1012–1021. 24696718 PMC3971304

[52] N. Cascavilla , P. Musto , G. D'Arena , et al., “CD117 (c‐Kit) is a Restricted Antigen of Acute Myeloid Leukemia and Characterizes Early Differentiative Levels of M5 FAB Subtype,” Haematologica 83, no. 5 (1998): 392–397. 9658721

[53] E. Takematsu , M. Massidda , J. Auster , et al., “Transmembrane Stem Cell Factor Protein Therapeutics Enhance Revascularization in Ischemia without Mast Cell Activation,” Nature Communications 13, no. 1 (2022): 2497, 10.1038/s41467-022-30103-2. PMC9076913 35523773

[54] J. Yoo , C.‐Y. Li , S. M. Slone , C. Maffeo , and A. Aksimentiev , A Practical Guide to Molecular Dynamics Simulations of DNA Origami Systems (Springer New York, 2018), 209–229. 10.1007/978-1-4939-8582-1_15 29926456

[55] J. Y. Lee , H. Koh , and D.‐N. Kim , “A Computational Model for Structural Dynamics and Reconfiguration of DNA Assemblies,” Nature communications 14, no. 1 (2023): 7079. 10.1038/s41467-023-42873-4 PMC10625641 37925463

[56] J. Řezáč , P. Hobza , and S. A. Harris , “Stretched DNA Investigated Using Molecular‐Dynamics and Quantum‐Mechanical Calculations,” Biophysical Journal 98, no. 1 (2010): 101–110. 20074515 10.1016/j.bpj.2009.08.062 PMC2800961

[57] L. Zhang , O. Lampela , L. Lehtiö , and A. H. Juffer , “Insights into the Behaviour of Phosphorylated DNA Breaks From Molecular Dynamic Simulations,” Computational Biology and Chemistry 115 (2025). 10.1016/j.compbiolchem.2024.108337 39752851

[58] M. T. Sykes and M. Levitt , “Simulations of RNA Base Pairs in a Nanodroplet Reveal Solvation‐Dependent Stability,” Proceedings of the National Academy of Sciences 104, no. 30 (2007): 12336–12340, 10.1073/pnas.0705573104. PMC1920539 17636124

[59] M. Zgarbová , M. Otyepka , J. Šponer , F. Lankaš , and P. Jurečka , “Base Pair Fraying in Molecular Dynamics Simulations of DNA and RNA,” Journal of Chemical Theory and Computation 10, no. 8 (2014): 3177–3189, 10.1021/ct500120v. 26588288

[60] G. Hancu and A. Modroiu , “Chiral Switch: Between Therapeutical Benefit and Marketing Strategy,” Pharmaceuticals 15 (2022): 240. 35215352 10.3390/ph15020240 PMC8877306

[61] A. Bajpai , P. K. Dwivedi , and S. Sivakumar , “Chiral Nanomaterial‐Based Approaches for Diagnosis and Treatment of Protein‐Aggregated Neurodiseases: Current Status and Future Opportunities,” Journal of Materials Chemistry B 12, no. 8 (2024): 1991–2005. 38333942 10.1039/d3tb02381h

[62] K. Agrawal , “Daunorubicin,” in xPharm: The Comprehensive Pharmacology Reference (Elseiver Publishing, 2007), 1–4.

[63] H. A. Blair , “Daunorubicin/Cytarabine Liposome: A Review in Acute Myeloid Leukaemia,” Drugs 78, no. 18 (2018): 1903–1910, 10.1007/s40265-018-1022-3. 30511323 PMC6314217

[64] E. Sheikh , T. Tran , S. Vranic , A. Levy , and R. D. Bonfil , “Role and Significance of c‐KIT Receptor Tyrosine Kinase in Cancer: A Review,” Bosnian Journal of Basic Medical Sciences 22 (2022): 683–698, 10.17305/bjbms.2021.7399. 35490363 PMC9519160

[65] S. Jiang , Z. Ge , S. Mou , H. Yan , and C. Fan , “Designer DNA Nanostructures for Therapeutics,” Chemistry (Weinheim An Der Bergstrasse, Germany) 7, no. 5 (2021): 1156–1179, 10.1016/j.chempr.2020.10.025.

[66] Q. Jiang , Y. Shang , Y. Xie , B. Ding , and D. N. A. Origami , “From Molecular Folding Art to Drug Delivery Technology,” Advanced Materials 36, no. 22 (2023). 10.1002/adma.202301035 37715333

[67] S. Ren , K. Fraser , L. Kuo , et al., “Designer DNA Nanostructures for Viral Inhibition,” Nature Protocols 17, no. 2 (2022): 282–326, 10.1038/s41596-021-00641-y. 35013618 PMC8852688

[68] C. Sigl , E. M. Willner , W. Engelen , et al., “Programmable Icosahedral Shell System for Virus Trapping,” Nature Materials 20, no. 9 (2021): 1281–1289, 10.1038/s41563-021-01020-4. 34127822 PMC7611604

[69] M. Mao , Z. Lin , L. Chen , et al., “Modular DNA‐Origami‐Based Nanoarrays Enhance Cell Binding Affinity through the “Lock‐and‐Key” Interaction,” Journal of the American Chemical Society 145, no. 9 (2023): 5447–5455, 10.1021/jacs.2c13825. 36812464

[70] L. Guo , Y. Zhang , Y. Wang , et al., “Directing Multivalent Aptamer‐Receptor Binding on the Cell Surface With Programmable Atom‐Like Nanoparticles,” Angewandte Chemie 134, no. 18 (2022). 10.1002/anie.202117168 35226386

[71] S. Umrao , A. Dwivedy , and X. Wang , “Engineering Advanced Functional Nanomaterials for Virus Detection,” in Nano‐Engineering at Functional Interfaces for Multi‐Disciplinary Applications (Elsevier, 2025), 445–472.

[72] N. Chauhan and X. Wang , “Nanocages for Virus Inhibition,” Nature Materials 20, no. 9 (2021): 1176–1177, 10.1038/s41563-021-01088-y. 34433933

[73] T. Song , J. Galván Achi , V. Anirudhan , et al., “Engineering Two‐Dimensional Nanobody‐Origami Architectures for Enhanced Antiviral Activity,” Nano Letters 25, no. 47 (2025): 16586–16592, 10.1021/acs.nanolett.5c03096. 41247251 PMC12874547

[74] S. Umrao , A. Dwivedy , D. Gandavadi , et al., “DNA Nanostructure‐Templated Multivalency Enables Broad‐Spectrum Virus Inhibition,” Advanced Science (2025): 13710, 10.1002/advs.202513710. PMC12970221 41270218

[75] X. Shi , A.‐K. Pumm , C. Maffeo , et al., “A DNA Turbine Powered by a Transmembrane Potential across a Nanopore,” Nature Nanotechnology 19, no. 3 (2023): 338–344, 10.1038/s41565-023-01527-8. PMC10950783 37884658

