# Supplementary material for: DNA Origami‐Templated Aptamer Chiral Structures Realize Cellular Enantioselectivity
Source: Adv Mater. 2026 Jan 16;38(12):e19007. doi: 10.1002/adma.202519007 (PMC12933013; doi:10.1002/adma.202519007)
Supplement: Supplementary file 1 — Supporting File: adma72153‐sup‐0001‐SuppMat.pdf. [file ADMA-38-e19007-s001.pdf]

Supplementary Information

DNA origami-templated aptamer chiral structures realize cellular enantioselectivity

Tingjie Song[1,2,3,#,*], Abhisek Dwivedy[1,2,4,#,*], Dhanush Gandavadi[4], Gayatri Chandran[5], Yiquan An[6], Mengxi Zheng[1,2,4], Xiaojing Wang[2,3], Lifeng Zhou[6], Yang Zhao[1,2,4,5,7], Xing Wang[1,2,3,4,6,*]

[1] Carl R. Woese Institute for Genomic Biology, University of Illinois at Urbana-Champaign, Urbana, IL 61801, USA

[2] Holonyak Micro and Nanotechnology Lab, Grainger College of Engineering, University of Illinois at Urbana-Champaign, Urbana, IL 61801, USA

[3] Department of Chemistry, University of Illinois at Urbana-Champaign, Urbana, IL 61801, USA

[4] Department of Bioengineering, Grainger College of Engineering, University of Illinois at Urbana-Champaign, Urbana, IL 61801, USA

[5] Department of Electrical and Computer Engineering, Grainger College of Engineering, University of Illinois at Urbana-Champaign, Urbana, IL 61801, USA

[6] Department of Advanced Manufacturing and Robotics, Peking University, Beijing, 100871, China

[7] Cancer Center at Illinois, University of Illinois at Urbana-Champaign, Urbana, IL 61801, USA

# Co-first authors: Tingjie Song, Abhisek Dwivedy

* Corresponding authors: Tingjie Song (tsn@illinois.edu), Abhisek Dwivedy (abhisekd@illinois.edu), Xing Wang (xingw@illinois.edu)

METHODS

Materials

The scaffold P7560 DNA was obtained from tilibit nanosystems. The staple DNA oligos were obtained from IDTDNA. The CD117 targeting aptamers (with and without 5-FAM conjugates) were commercially synthesized from IDTDNA. The sequence of the aptamer is protected under an US provisional patent. These DNA molecules were used in this study without further purification. The cells Kasumi-1, HL-60, HEL9.1.7, Ramos and Raw 264.7 were procured from ATCC. Cell culture medium RPMI-160, heat inactivated Fetal Bovine Serum, 100X antibiotic-antimycotic solution, TrypLE-Express, Lysotracker Red, MTT kit were procured from Thermo Fisher.

Molecular docking and generation of protein-aptamer complex

The 3D structure for CD117 was retrieved from Protein Data Bank (PDB ID: 2E9W)[1]. The 3D structure of the CD117 DNA aptamer was generated from its sequence using the 3D RNA webserver[2]. The aptamer was docked on to a protein monomer using the Haddock 2.4 webserver[3]. The dimer complex consisting of two aptamers, each bound to a respective protein monomer was generated using Pymol 3.1[4]. The resultant structure was exported and further minimized using the Refinement module of the Haddock 2.4 webserver[3]. For integration of this structure on the all-atom CAP model in oxDNA, bonds were created between amino acid residues from the protein and nucleotides from the aptamer using Pymol 3.1 and the resultant structure was further refined using the Haddock 2.4 webserver. The participating atoms in the artificially created covalent linkages were selected based on valence (selecting atom attached directly to hydrogens) and distance criteria between the atoms satisfying covalent (carbon-carbon, 1.54 Å) (4 Å) interactions. The residues for bond creation were chosen from the major hydrophobic interaction present between the aptamer and the protein.

Generation of all atom CAP, protein-CAP models and coarse-grained simulations

The DNA nanorods were designed using caDNAno[5], including the L, R half pitch, achiral designs. In addition, the protein-aptamer dimers were assembled onto the DNA nanorods within Chimera[6]. The coarse-grained simulations of the DNA tubes were conducted by using oxDNA[7]. The caDNAno files of DNA tubes were double-checked by using oxView[8] and the simulation files of oxDNA were exported from the oxView website, including the topology and starting configuration files. Then, the simulations were finished by following the standard procedure and conditions of oxDNA[7]. First, perform an initial energy minimization for 20,000 steps. Second, relax the structure for 20,000 steps at 300 K using a Langevin thermostat with a diffusion coefficient of 2.5. Finally, conduct a Monte Carlo simulation for 100,000 steps. All simulation results were visualize within oxView and analyzed by using oxDNA analysis tools[7].

Energy minimization of protein-CAP models

While all atom energy minimization and molecular dynamics simulations of DNA nanostructures have been reported in the past, such protocols haven't been developed for DNA nanostructures in complex with multiple proteins. To circumvent this, we developed a protocol for all-atom energy minimization of the protein-CAP models using the AMBER force fields ff19SB-BSC1-OPC for protein and nucleic acid components and the Generalized AMBER Force Field for small molecules and ions. The 3D structures of the protein-CAP models were examined to remove incomplete residues and steric clashes, and the artificially created covalent bonding between aptamers and proteins were verified. The protein-CAP models were solvated using the TIP3P water model with addition of sodium, chloride and magnesium for neutralization in a 30 Å shell. Use of a cubic cell was avoided owing to greater computational requirements. Short- and long-range electrostatic interactions were calculated using Lennard Jones and Particle Mesh Ewald constraints, respectively, with cut-off 8 and 2 Å, respectively. To preserve the tubular geometry of the DNA origami during relaxation, positional restraints were applied to the scaffold and staple strands, treating the tube as a static framework. Only the aptamers and protein components were allowed to move during energy minimization. Restraints were implemented using a harmonic potential with a force constant of 500 $kcal \cdot mol^{-1} \cdot Å^{-2}$ applied to all non-hydrogen atoms of the tube. Energy minimization was conducted for 5000 steps, comprising 3000 steps of steepest descent followed by 2000 steps of conjugate gradient, under constant volume conditions (1 bar pressure, 310 K) with nonbonded interaction cutoff of 10 Å. To evaluate the thermodynamic impact of minimization, molecular mechanics Poisson–Boltzmann surface area (MM/PBSA) calculations were performed. Free energy estimates were computed before and after minimization using including molecular mechanics energy, polar and non-polar solvation energy. Representative structures were stored per 1000 steps of minimization and visualized using UCSF Chimera X for comparative analysis.

Tubular-shaped DNA origami nanostructures assembly

The DNA origami tubes were folded using a P7560 scaffold and ten times the concentration of staple strands (**Table S1** and **S2**). The mixture was dissolved in a 1× TAE buffer with 14 mM magnesium acetate. The assembly process was carried out in a Bio-Rad Thermal Cycler, starting with a heating step at 80 °C for 5 minutes. The samples were then cooled to 60 °C at a rate of 1 °C per 5 minutes, followed by cooling from 60 °C to 35 °C at a rate of 0.5 °C per 45 minutes. Finally, the program cooled the samples to 15 °C at a rate of 1 °C per 5 minutes.

DNA tubes purification

To get rid of the excess of staple strands, the DNA tubes were purified with 1% agarose gel

with 1× SYBR Green I. After the running of the gel in 0.5× TAE with 11 mM magnesium acetate, the targeted band was cut out and the DNA tubes were extracted from the gel with Freeze 'N Squeeze spin columns (BioRad). The concentration of the DNA tubes was then measured using a NanoDrop UV-Vis spectrophotometer (Thermo Fisher).

Synthesis of 13 nm gold nanoparticle

The 13 nm gold nanoparticles were synthesized according to the previous protocol.[9] First, 100 mL of 1 mM $HAuCl_4$ was boiled with stirring, which was followed the addition of 10 mL sodium citrate (38.8 mM). The heating was maintained for an additional 10 minutes before the oil bath was removed. After the solution cooled to room temperature, it was filtered using a 0.45 μm filter. The stock solution of gold nanoparticles was stored at 4 °C for future use.

ssDNA-gold nanoparticle conjugation

The thiolated linker strands and spacer strands (**Table S3**) were mixed in a 1:1 ratio. Then, the DNA modification procedure was performed as the reported protocol.[10] 350-fold excess of the above mixture was added to the bare gold nanoparticle solution and incubated at 4°C for 12 hours. Afterward, the pH of the solution was adjusted using 100 mM phosphate buffer (pH 7.4), followed by gradually increasing the NaCl concentration to 0.3 M at a rate of 0.1 M every 8 hours. The resulting DNA-modified gold nanoparticles were purified by centrifugation at 13,000 rpm for 25 minutes, repeated three times.

Immobilization of gold nanoparticles on DNA tubes

The purified DNA tubes were mixed with a 50-fold excess of gold nanoparticles in 1× TAE buffer containing 11 mM magnesium acetate. The reaction was incubated for 5 days. The mixture was then purified using 0.8% agarose gel electrophoresis. The desired band was excised and extracted using Freeze 'N Squeeze spin columns.

Atomic force microscopy (AFM) imaging

AFM imaging was performed using the Asylum Research Cypher Atomic Force Microscope. All scans were conducted in air mode. The AFM tips (OTESPA-R4) were purchased from Bruker. Fresh mica (Ted Pella) was used for sample preparation.

Transmission electron microscopy (TEM) imaging

TEM imaging was performed using the JEOL 2100 CRYO TEM operating at 200 kV. Quantifoil TEM Substrate grids (668-200-CU) were purchased from Ted Pella. For the imaging, negatively stained samples were prepared with 2 % aqueous uranyl formate solution.

Synthesis of chiral aptamer pattern (CAP) constructs

The 54-fold CD117 aptamers (Table S4) were mixed with purified DNA nanotubes and kept at 4°C for 24 hours. Unbound free aptamers were removed using 100 kDa Amicon Ultra centrifugal filters. The concentrations of the resulting nanostructures were measured with the Nanodrop and then stored at 4°C for further use.

Single-molecule localization microscopy- sample preparation

A 2nM sample of DNA origami chiral nanostructures was prepared in 12mM magnesium acetate solution. 50uL of this solution was flushed into a flow chamber. The flow chamber was constructed by placing a clean #1.5 coverslip on two pieces of double-sided tape (2.4 mil thick) on a glass slide. The coverslip was cleaned prior to this experiment by immersion in 1M KOH and sonication for 15 mins, followed by 2-3 washes and drying with compressed air. The flow chamber thus constructed was filled with the solution containing nanostructures and allowed to settle for 2 mins. This ensures that a fraction of the nanostructures stick to glass and remain within the focus plane during imaging. After 2 mins incubation, the chamber was flushed three times with a freshly prepared imaging buffer solution that consists of 10% glucose, 1% glucose oxidase and β-mercaptoethylamine (MEA, or cysteamine) dissolved in 1x PBS (phosphate buffer solution). The imaging buffer solution facilitates Cy5's blinking behavior when exposed to red laser (~647nm wavelength) of sufficient power density[11, 12].

STORM - optical setup

The optical path for stochastic optical reconstruction microscopy (STORM) imaging[13, 14] of the chiral nanostructures is configured for TIRF-M (Total Internal Reflection Fluorescence Microscopy)[15, 16]. The custom setup is built around an inverted Olympus IX81 microscope. The light beam from a 637nm wavelength diode laser (Coherent, OBIS LX) is expanded (10x), collimated and focused onto the back focal plane of a 100X TIRF objective (1.49 NA, Olympus UAPON 100XOTIRF). The focusing lens is installed on a motorized translational stage (Zaber technologies) near the microscope's back port. The stage can offset the focused beam from the objective's optical axis – thus changing the angle of incidence at the glass-water interface. An offset > 2.5mm from EPI-fluorescence shifts the incidence angle beyond the critical angle – achieving total internal reflection at the sample plane. The microscope's filter turret is equipped with a Cy5 filter cube (Edmund optics excitation filter #67-035, dichroic filter #67-084). The 637 nm excitation beam reaches the sample and the emission from Cy5 molecules is collected through the objective and an emission filter (Edmund optics #67-038) and focused onto an sCMOS camera sensor (Photometrics Prime BSI).

STORM - image acquisition

In the presence of the imaging buffer, the red laser switches a large fraction of the Cy5 molecules in the exposed field of view to a 'dark' state. This decreases the density of fluorescent emitters in the field of view. At any point in time, a few molecules will stochastically transition into the excited state and return to the non-fluorescent dark state after emission[17]. By acquiring long acquisitions from a field of view, the image can be reconstructed by localizing and overlaying isolated individual emitters. TIRF illumination rejects background fluorescence, improving the signal-to-noise ratio of emitters on the focus plane. The camera is operated at 50Hz frame rate, 100 MHz readout speed, 0.93 gain and has an RMS noise of 1.7 electrons. 20000 frames were acquired from each field of view that was imaged. Laser power density at the sample plane (at EPI) was maintained at ~10 μW/μm2 during data acquisition.

STORM - post processing

The raw image stacks were post processed using the ImageJ ThunderSTORM plugin[18]. The effective pixel size is 65nm (sample is magnified 100x on camera sensor pixels that are 6.5μm in length). For sub-pixel localization of Cy5 labels, background noise was minimized using a wavelet filter and local maxima detected for identifying approximate locations of emitters. The sub-pixel localization was performed by maximum likelihood fitting. The final results were drift-corrected and filtered based on density of localizations to isolate labelled nanostructures present at the focus plane. Localized Cy5 labels were rendered using normalized Gaussian functions. Super-resolution image reconstruction was also performed in Insight3 software, generously provided by Dr. Bo Huang (University of California, San Francisco). The average localization precision is ~7nm.

Surface plasmon resonance (SPR) assay

CD117 proteins (Avantor, Inc.) were conjugated on SPR spectroscopy CM5 sensor chips (Cytiva) through the EDC/NHS reaction. The running buffer was 20 mM HEPES buffer with 150 mM NaCl and 0.05% Tween-20. The binding assays were tested on the Biocore T200 SPR system. The kinetics were evaluated at the 1:1 binding model.

Cell maintenance and experimental setups

The cells were maintained in RPMI-1640 medium supplemented with 10% Fetal Bovine Serum and 1X antibiotic-antimycotic solution and incubated in a CO_2 incubator at 37°C and 5% CO_2. Medium was replenished every 3 days and cells were passaged once every week. Kasumi-1, HL-60, HEL9.1.7, and Ramos were passaged by centrifugation at 300xg for 10 minutes and harvesting the cells. For Raw264.7, the medium was removed, and the cells were treated with TrypLE-Express for 10 minutes at 37°C and the cells were harvested by centrifugation as described earlier. For experiments, at least 3 independently grown cultures were used as

replicated. Before experimental setup, the cells were harvested, resuspended in a buffer (20 mM HEPES pH 7.2, 150 mM NaCl, 12.5 mM $MgCl_2$ and 10% FBS) counted using trypan blue staining in a Thermo Countess 2 cell counter. Approximately 10^4 cells were used per replicate per conditions in each experiment. The cells were treated with 14 ng/ul chiral L and R nanoparticles, chiral L and R nanotubes carrying the folded CD117 aptamers (FAM-conjugated) for 1 and 2 h. Untreated cells served as controls. 30 minutes prior to the end of treatment, lysotracker red was to the cells. Cells were washed thrice at the end of the treatment by centrifugated at 300xg for 10 minutes and resuspended in the buffer for flow cytometry and confocal analysis.

Flow cytometry assay

Flow cytometry was performed using a Thermo Attune flow cytometer equipped with blue (488 nm) and red (637 nm) lasers and 96-well plate CytKick autosampler. The data acquisition was performed using the Attune Cytometric Software V5.1.1. The cells were first gated on Forward Scatter Area (FSC-A) and Side Scatter Area (SSC-A) to select the live cells for analysis. The live cells were further gated on Forward Scatter Area (FSC-A) and Forward Scatter Height (FSC-H) to select the singlets. The singlets were then analyzed for FAM signals using the band pass filter BL1 (530/30). The flow cytometry data was analyzed using the FCS Express Software V7.0.

Confocal microscopy imaging

Confocal microscopy images were taken on the Leica SP8 UV/Visible Laser Confocal Microscope. The FAM and LysoTracker Red were excited with 488nm and 561 nm lasers, respectively. 63x/1.40 HC PL APO Oil CS2 objective was used in the experiments. The confocal experiment data was analyzed with software ImageJ.

Daunorubicin loading on aptamers

The CD117 aptamers were loaded with daunorubicin following our previously reported protocol[19]. Daunorubicin intercalates in the duplex region of the DNA aptamer. Briefly the FAM-free CD117 aptamers (100 μM) were folded and subsequently incubated with 1.5 mM Daunorubicin for 24 hours in a rotator at room temperature. Excess unbounded daunorubicin was washed away by passing the mixture through a mini centrifugal filter unit (molecular weight cut-off 3 kDa). These daunorubicin loaded aptamers were then attached to the chiral nanotubes.

Cytotoxicity assays

Cells were harvest and distributed in 96-well plates as described above and treated with chiral

L and R nanotubes carrying the folded CD117 aptamers, chiral L and R nanotubes carrying the daunorubicin loaded folded CD117 aptamers. Cell treated with 10% DMSO and untreated cells served as positive and negative controls respectively, while concentration matched free daunorubicin served as internal control. 6 h post treatments, cells were washed by centrifugation and supplemented with fresh medium. 24 h post treatment, MTT dissolved in 1X PBS (pH 7.2) was added to the cells. 27 h post treatment, 2% v/v DMSO in 10% w/v acidified SDS was added to the cells. 30 h post treatment, the color development was observed, and the absorbance was recorded using a plate-reader spectrophotometer (Molecular Devices, USA). The absorbance data was normalized and converted to cell viability with respect to untreated sample. The viability data was plotted using GraphPad Prism V6.0.

SUPPLEMENTARY NOTE 1

Our review of CD117 (c-Kit) biology shows that the receptor exists as monomers on the cell surface, and activation begins when dimeric SCF binds two independent CD117 molecules, bringing their extracellular Ig-like domains 4 and 5 into the correct orientation for dimerization. Following this initial event, activated CD117 complexes redistribute into larger surface clusters through receptor patching and capping, a process in which additional free receptors migrate toward the activated units within the fluid membrane. These higher-order clusters enhance signaling efficiency and are ultimately internalized through endocytosis to prevent overstimulation[20-25].

Integrating these principles with our experimental and computational findings, we propose that the L-handed aptamer arrangement uniquely enables this natural sequence of events. The spatial placement of consecutive aptamers on L-CAP energetically favors CD117 dimerization, which then triggers local clustering and recruitment of additional monomers, leading to multiple dimer formations on the same L-CAP scaffold that eventually culminates in internalization of the L-CAP–CD117 complex. In contrast, R-CAP can bind CD117 monomers but does not favor dimerization, resulting in only transient interactions without clustering or cell uptake. A visual schematic of the putative mechanism for L-CAP interaction with CD117 and eventual internalization is presented in **Fig. S18**.

Thus, the chiral arrangement of aptamers on L-CAP not only initiates CD117 dimerization but also drives the downstream clustering and endocytosis pathway.

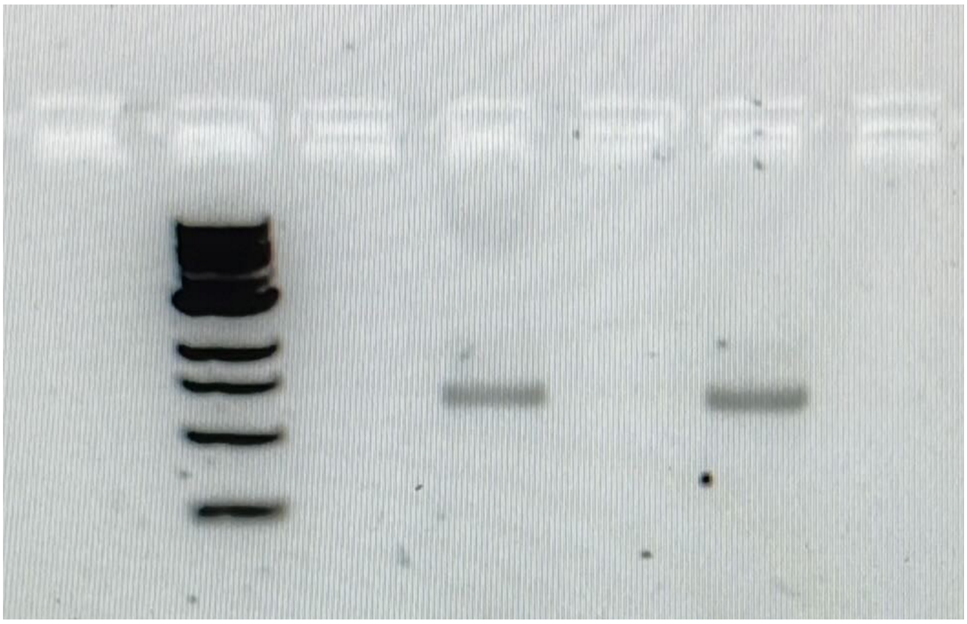

Figure S1: Characterization of L- and R-handed CAP constructs using agarose gel electrophoresis (AGE). Successful formation of the CAP constructs is analyzed by AGE. Left-lane: 1kb DNA ladder from NEB; Middle-lane: L-handed CAP construct; Right-lane: R-handed CAP construct.

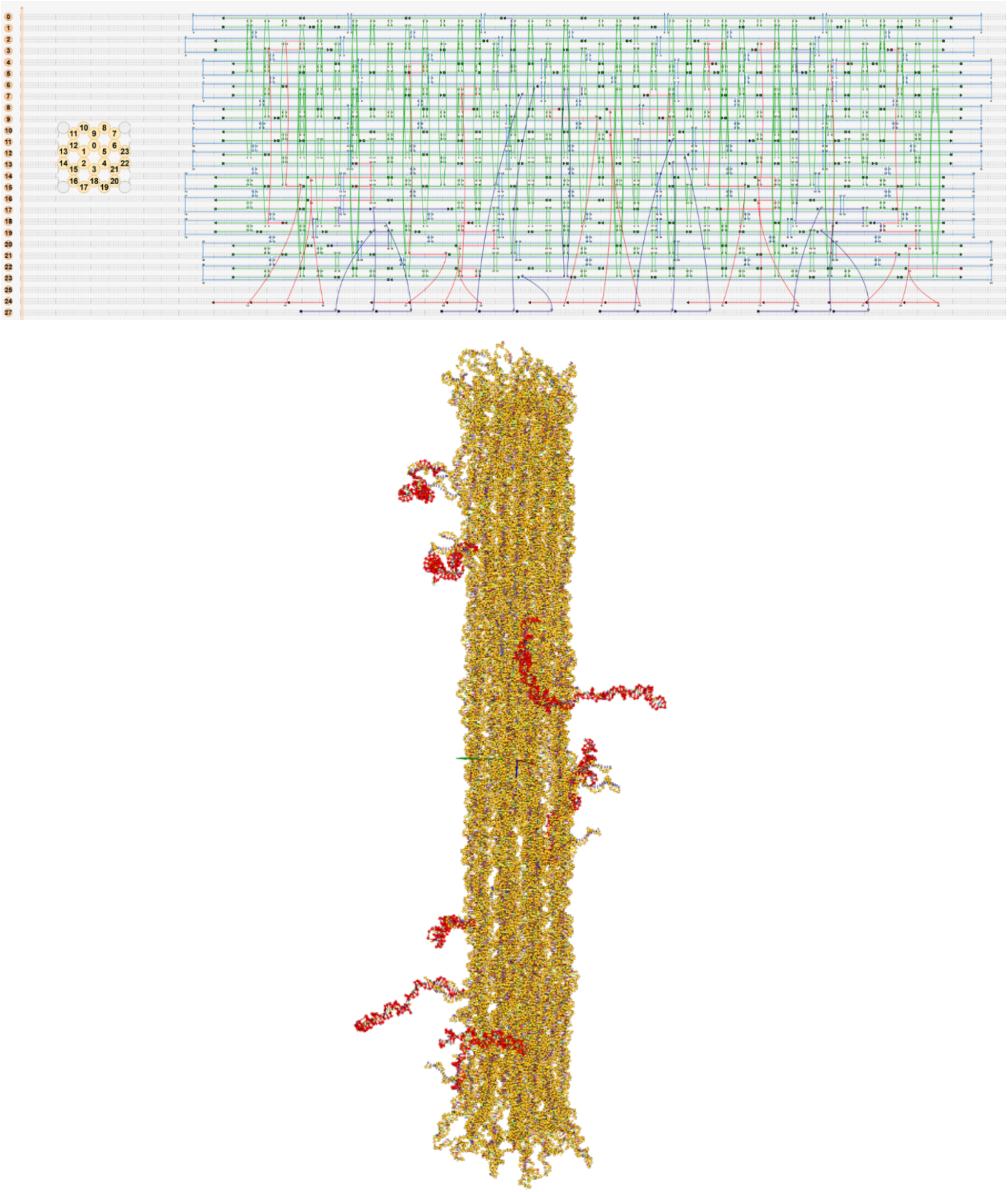

Figure S2: Top Panel: CADNano Map for Left-handed design. The distance between two adjacent aptamers is 9.5 nm. **Bottom Panel: oxDNA simulation screenshot of L-handed CAP construct**. Tubular-shaped DNA origami scaffold is displayed in brown color. The CD117-binidng aptamers docked on the DNA tube surface are shown in red.

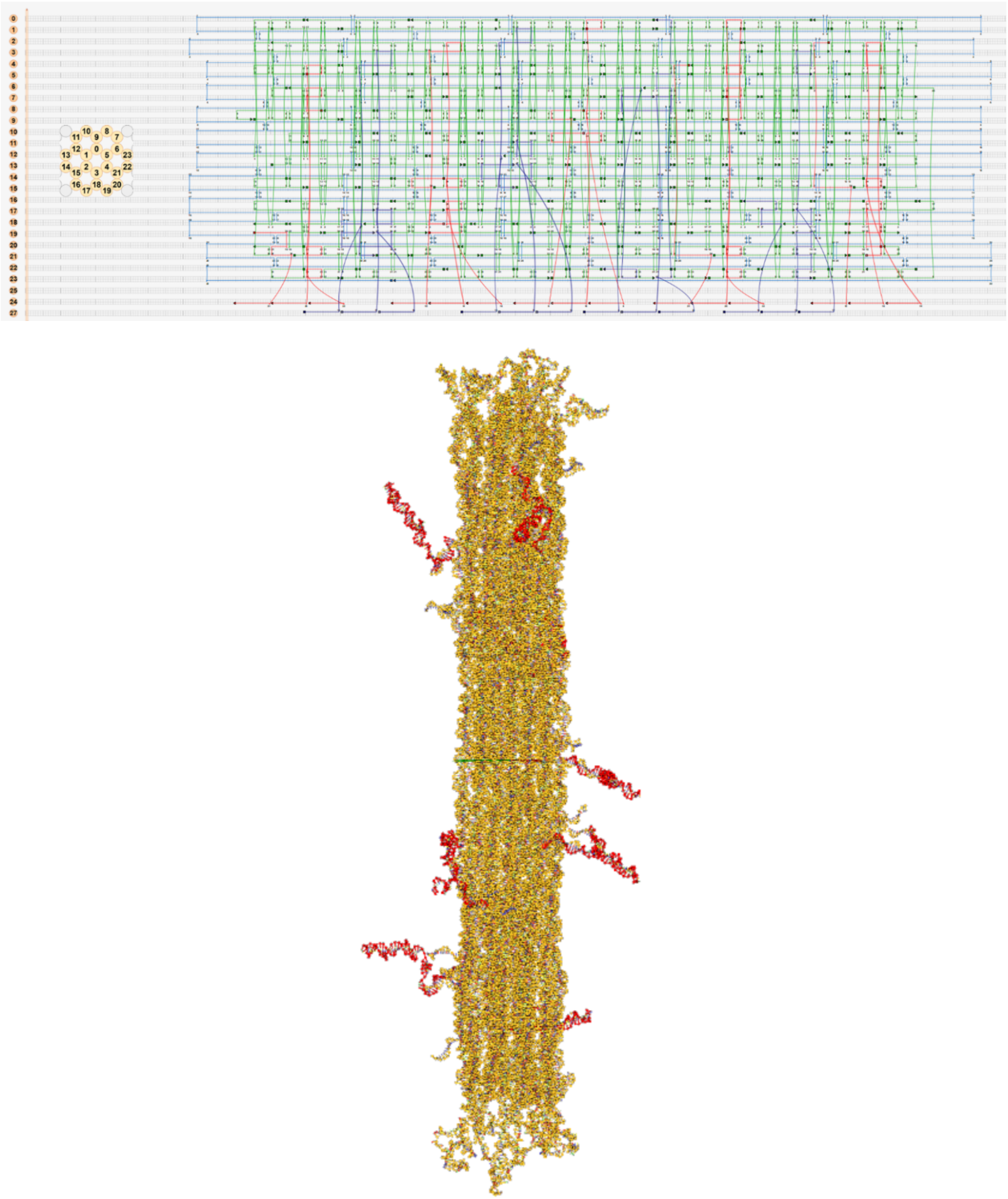

Figure S3: Top Panel: CADNano Map for Right-handed design. The distance between two adjacent aptamers is 9.5 nm. **Bottom Panel: oxDNA simulation screenshot of R-handed CAP construct**. Tubular-shaped DNA origami scaffold is displayed in brown color. The CD117-binidng aptamers docked on the DNA tube surface are shown in red.

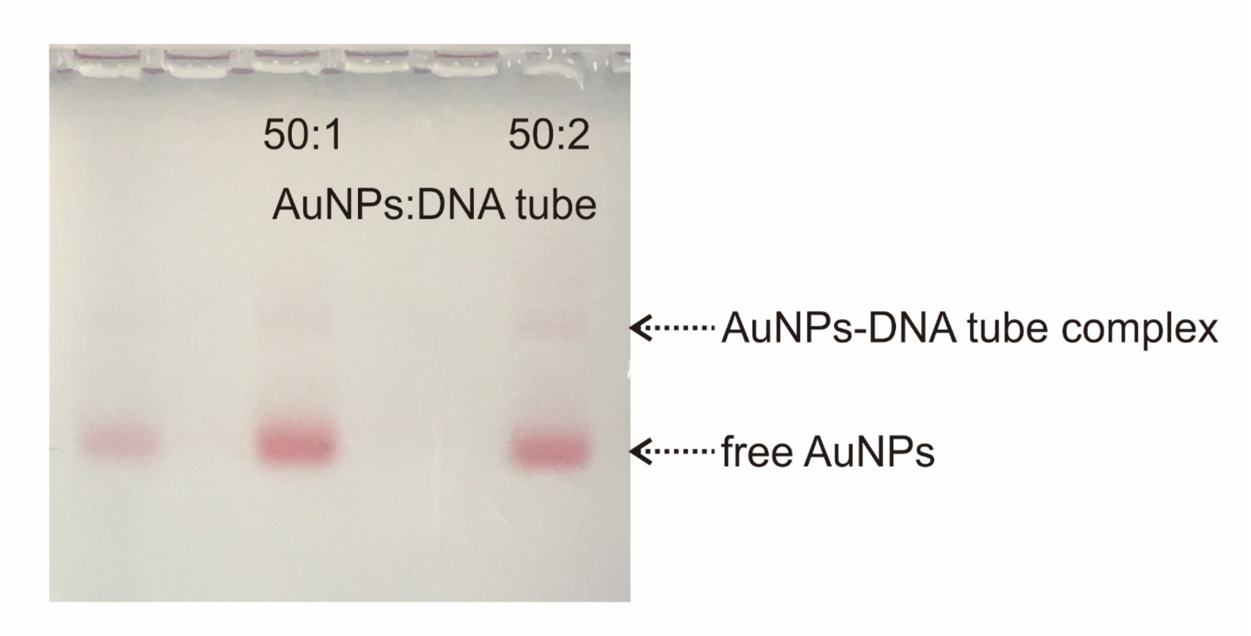

Figure S4: Characterization of L-handed DNA tube-AuNP complex using agarose gel electrophoresis (AGE) before complex purification to remove the free AuNPs. The upper band indicates the assembled DNA tube-AuNP complex, and the bottom band includes the free AuNPs not conjugated to the DNA tube. Left lane includes free AuNPs.

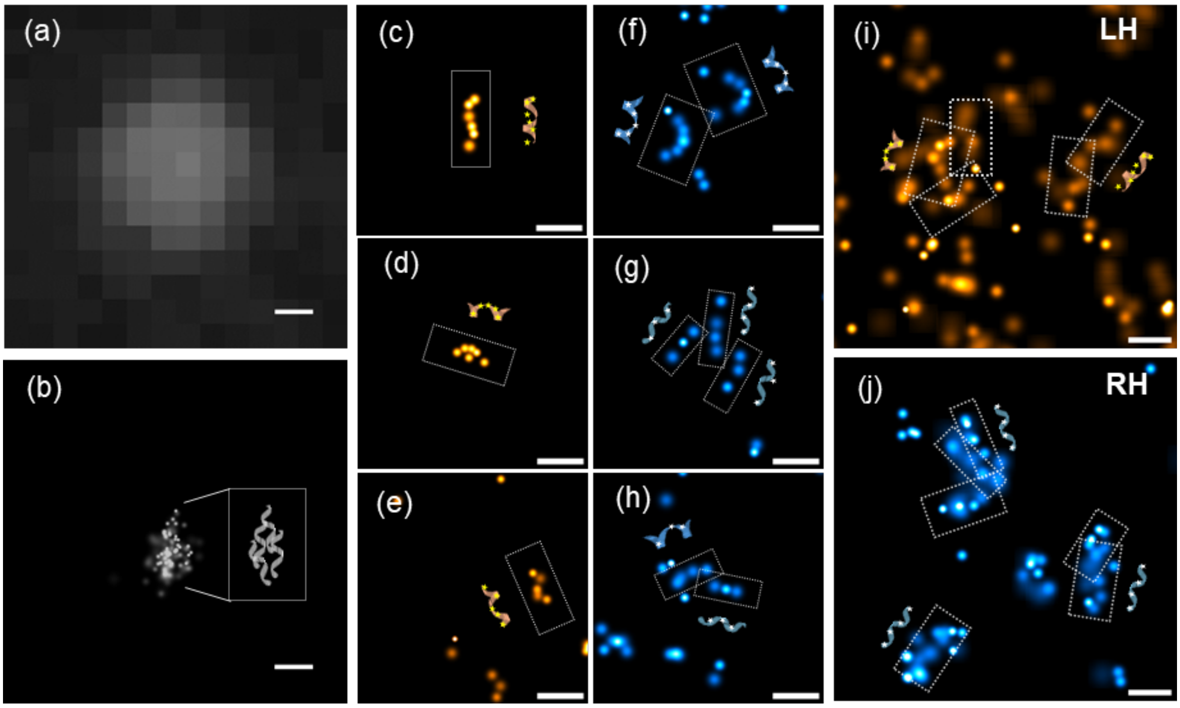

Figure S5. Super-resolution images of DNA origami twisted helical structures labelled with Cy5 spaced 10nm apart. The DNA structures were imaged in a flow chamber with ~5 nanostructures / μm2 density to avoid overcrowding. The Cy5 molecules were photo-switched to a dark state using 647 nm laser. They were allowed to thermally re-activate to perform single-molecule localization. Isolated fluorescent Cy5 molecules were localized via Gaussian fitting. These coordinate points were overlaid to form a reconstruction of the helical structures. (a) Diffraction-limited conventional image. (b) Super-resolution image of a cluster of the chiral nanostructures after post-processing raw data. Inset shows a graphical representation of a cluster of chiral structures, drawn to scale. (c,d,e) Reconstructed left-handed (LH) helices and side-by-side drawing of the labelled structure. (f,g,h) Right-handed (RH) helices. (i) LH nanostructures in a field of view of 0.8 μm. (j) RH particles in a field of view of 0.8 μm. Scale bars are 100 nm.

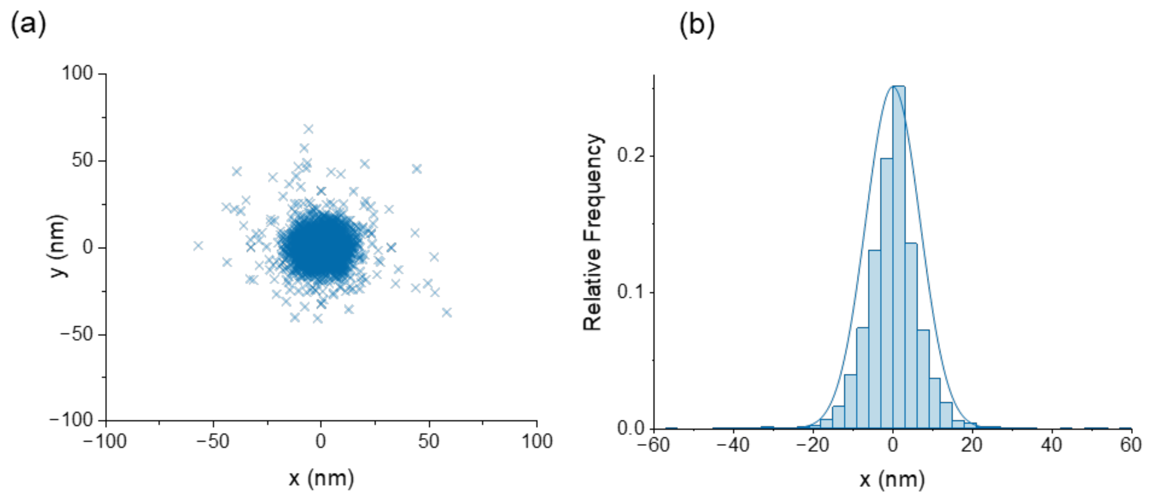

Figure S6. Calculation of the localization precision of STORM. (a) Multiple blinks from ~500 isolated Cy5 molecules were localized and overlayed on their centroids. (b) A histogram and Gaussian fit along x direction. The precision of localization is ~7nm.

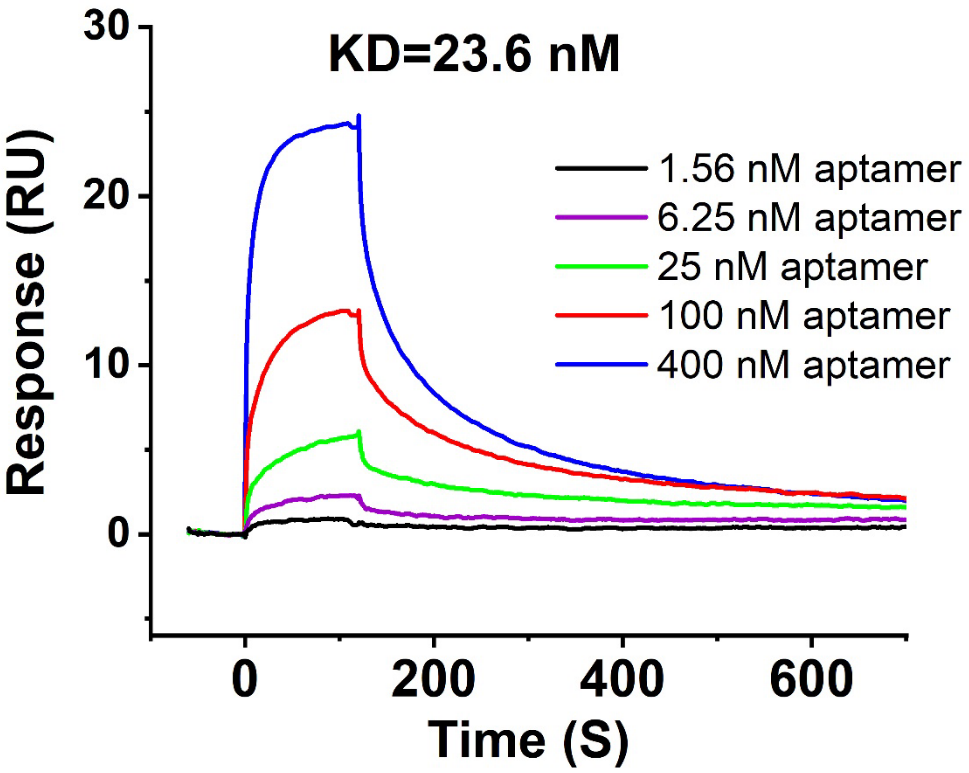

Figure S7: Surface plasmon resonance (SPR) analysis. SPR sensorgram of the interaction between CD117 protein with its binding aptamer at different concentrations. Each sensorgram is repeated three independent times with similar results and corrected for non-specific interaction of aptamers with negative control. The resultant sensorgrams were employed to determine binding kinetics parameters, including the association rate constant (k_a), dissociation rate constant (k_d), and binding equilibrium dissociation constant (K_D; $K_D = k_d/k_a$) as 23.6 nM.

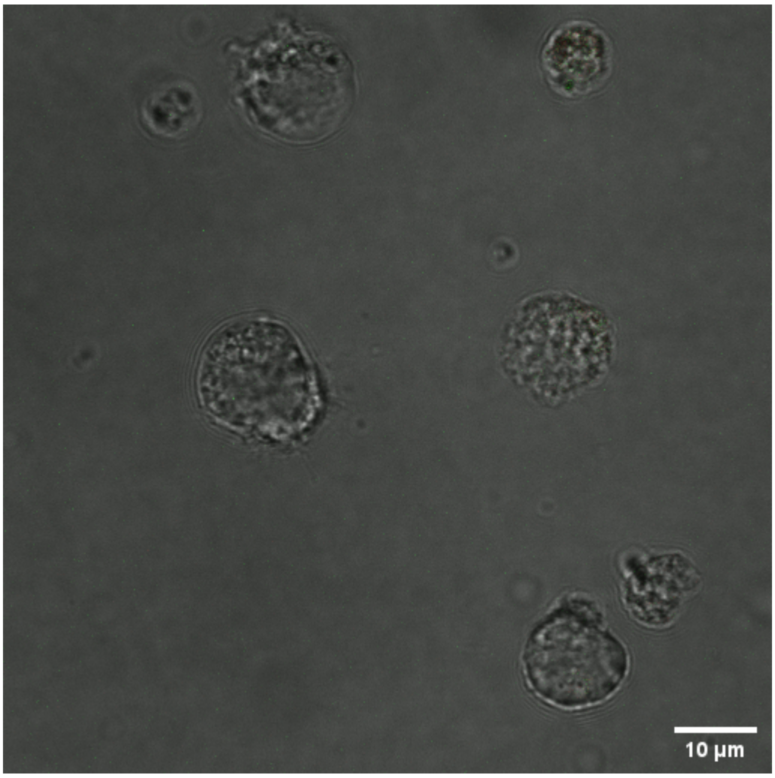

Figure S8: A confocal microscopy image of H9 cells at 2-hour incubation the monomeric CD117-binding aptamer.

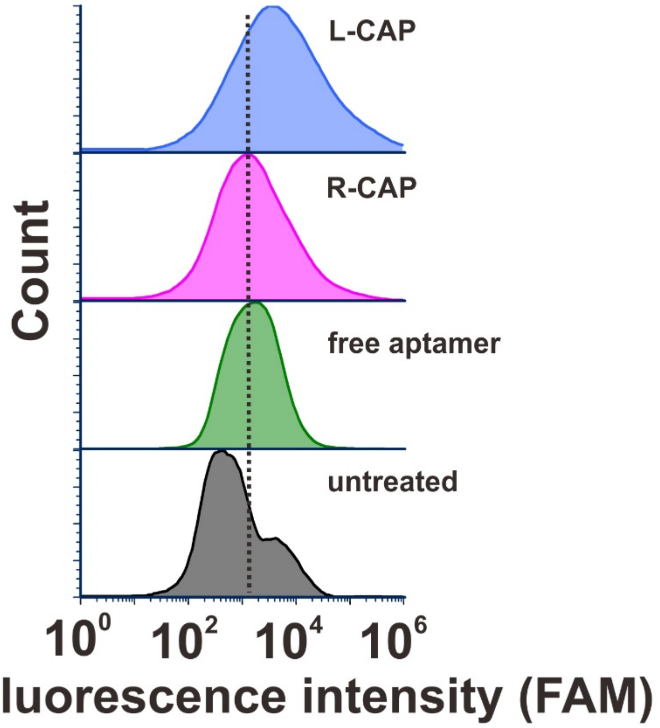

Figure S9: Comparative flow cytometry analysis of H9 cells interaction respectively with L-CAP, R-CAP, and free monomeric aptamer with a same, normalized aptamer concentration among all three samples.

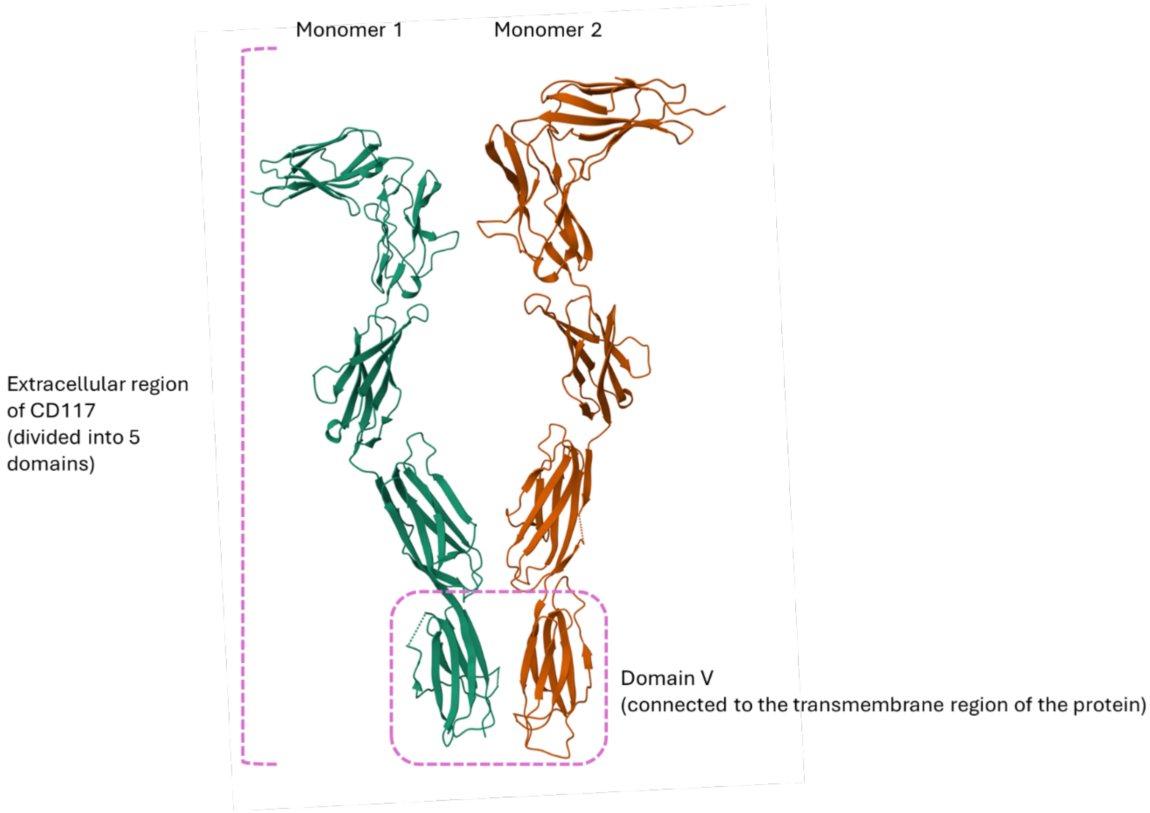

Figure S10: Crystal structure of extracellular region of the CD117 protein in its dimeric state showing its immunoglobulin like domains that facilitate its dimerization upon ligand binding. CD117 monomers and are functionally inactive and get activated following binding with the SCF (stem cell factor) dimer as its natural ligand. The binding of SCF-dimer results in dimerization of CD117 which initiates various downstream cell signaling[26]. The binding of CD117 to a non-natural ligand such antibodies or anti-CD117 compounds results in the internalization of the monomeric or dimeric forms. This internalization activates caspase mediated apoptosis resulting in cell death. Drug such as dasatinib and radotinib induce this internalization to clear AML[27]. Similarly, antibody-drug conjugates cause dimerization and internalization of CD117 to induce cell death in CD117 positive stem cells[28].

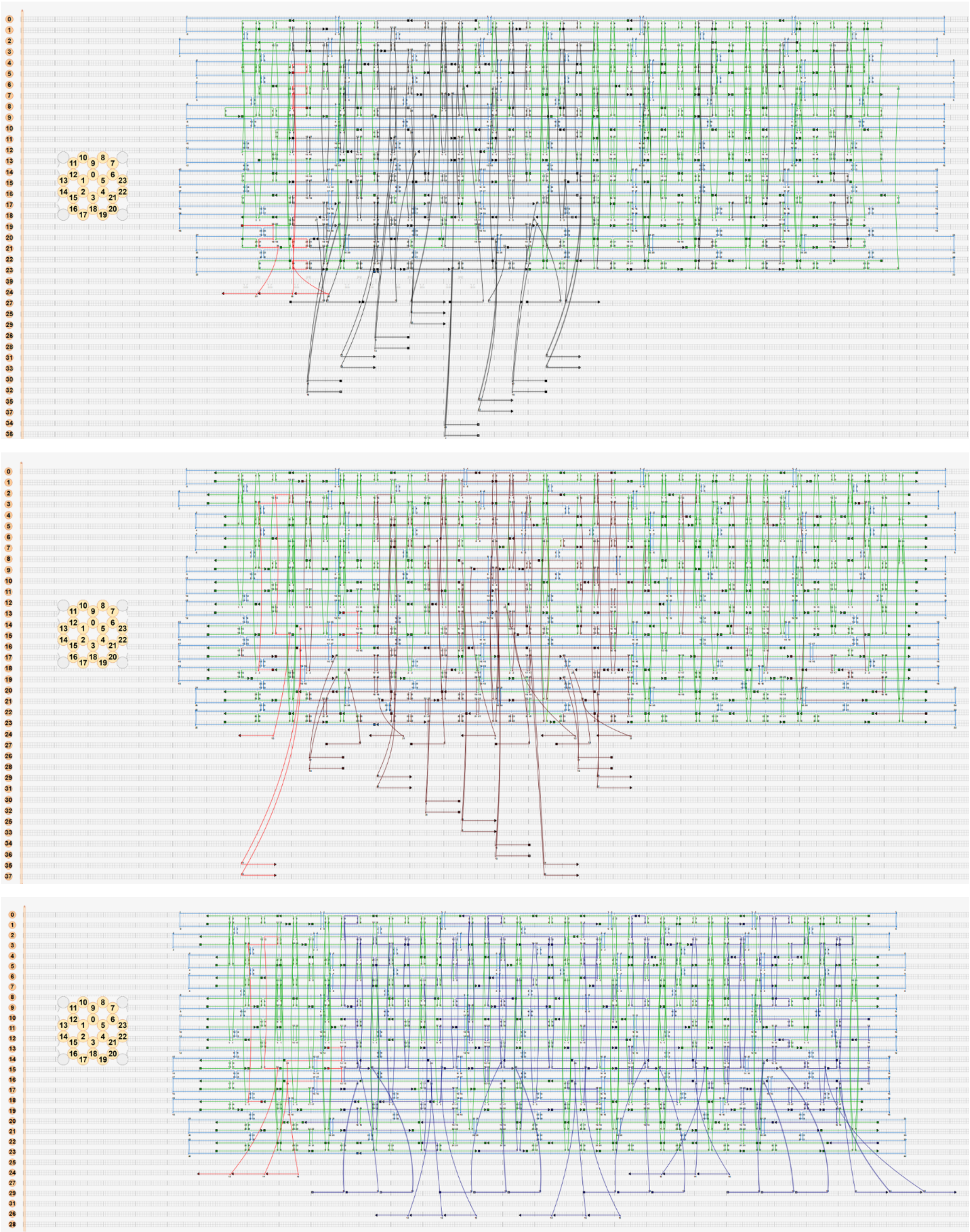

Figure S11. CADNano Lattice and Map for Half Pitch and Achiral DNA Nanotube Designs: **Top Panel-** Right-handed half pitch, distance between two adjacent aptamers is 4.75 nm. **Middle Panel-** Left-handed half pitch, distance between two adjacent aptamers is 4.75 nm. **Bottom Panel-** Achiral design, distance between two adjacent aptamers is 9.5 nm.

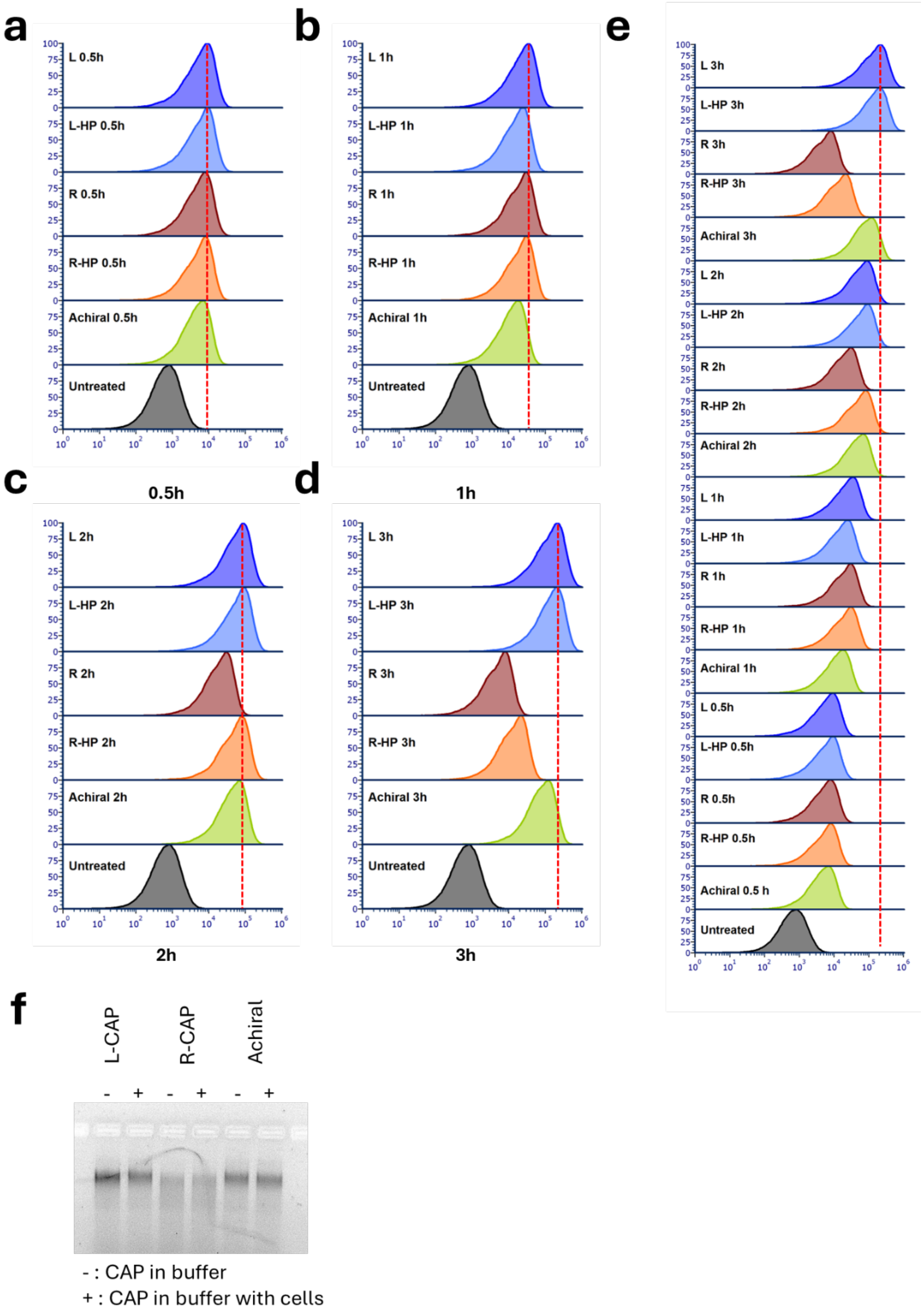

Figure S12: Flow cytometry histogram peak comparison of all 5 designs at (a) 0.5 h. (b) 1 h. (c) 2 h. (d) 3 h. (e) all time points. Red dashed lines represent the maximum fluoresce peak per plot. Each histogram is a representative of 6 independent replicates. (f) Gel electrophoresis profiles for L-CAP, R-CAP and Achiral structure incubated in buffer with out with cells reveal no cell or buffer mediated loss of structural integrity of the CAPs.

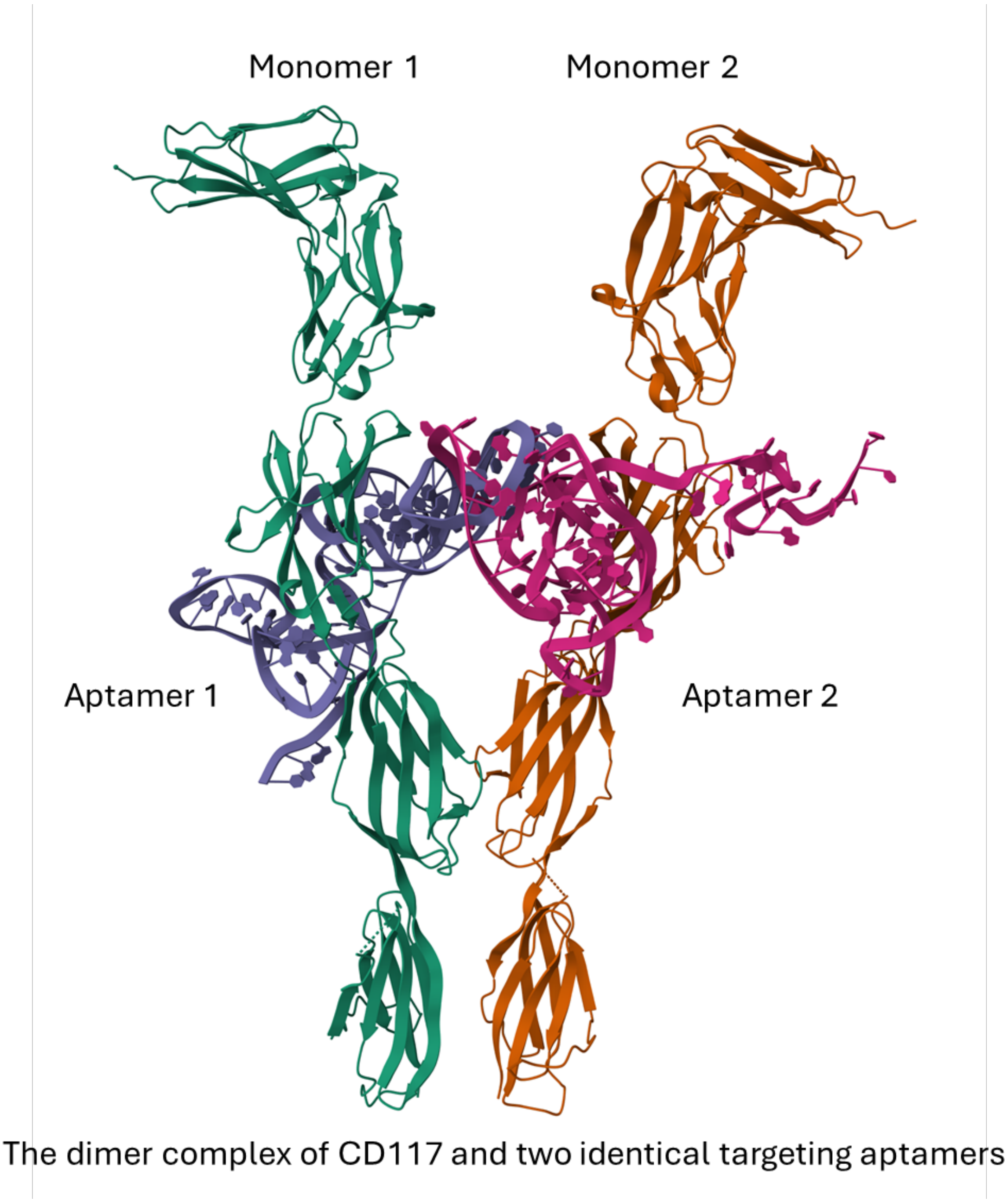

Figure S13: The dimer complex model of CD117 and two interacting aptamers. CD117 exists in the presented configuration in its physiological state. Each aptamer interacts with domains 3 and 4 of the interacting CD117 monomer without disrupting the dimerization of the protein.

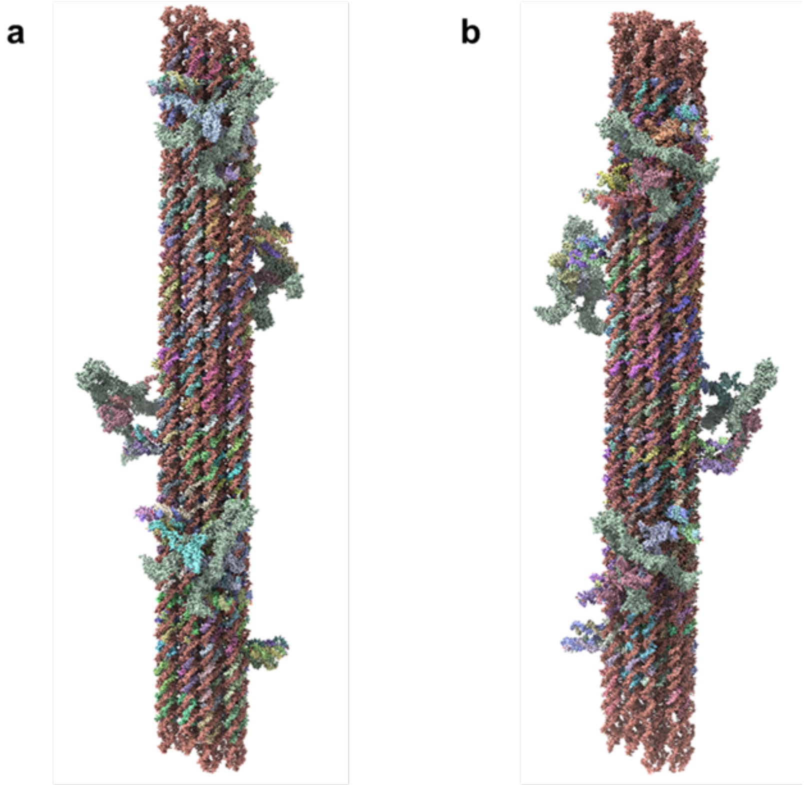

Figure S14. **DNA nanorods assembled with aptamer+protein dimers: (a)** left-handed and **(b)** right-handed.

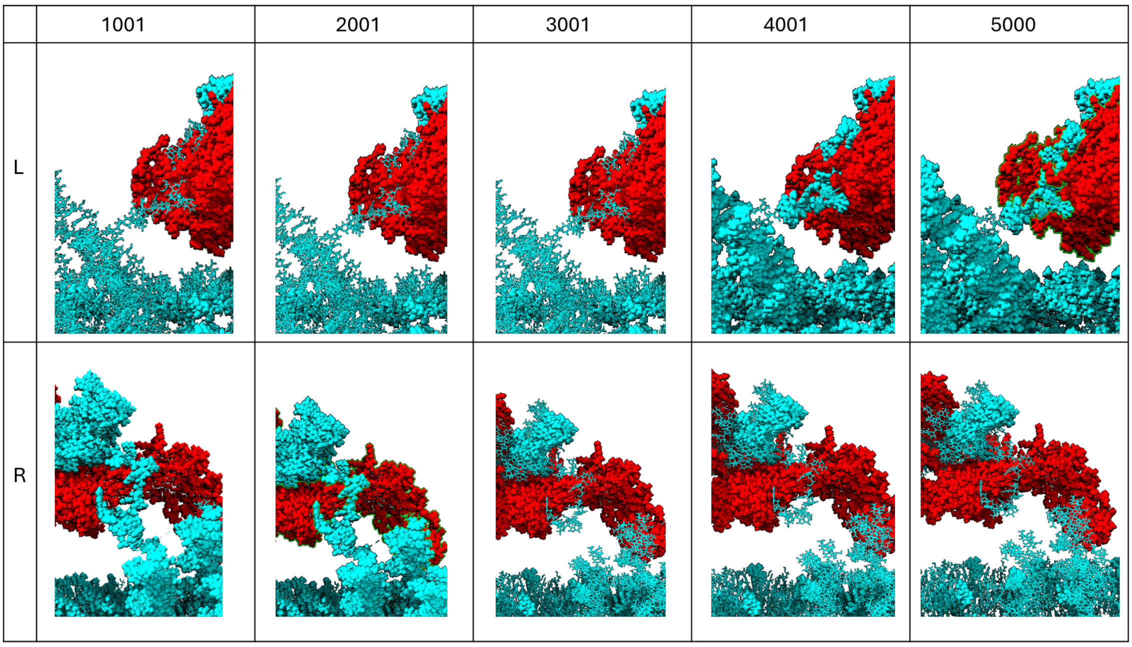

Figure S15: Point of view reveals the changes at an aptamer-dock site on L- vs R-CAP models across the 5000 steps of energy minimization.

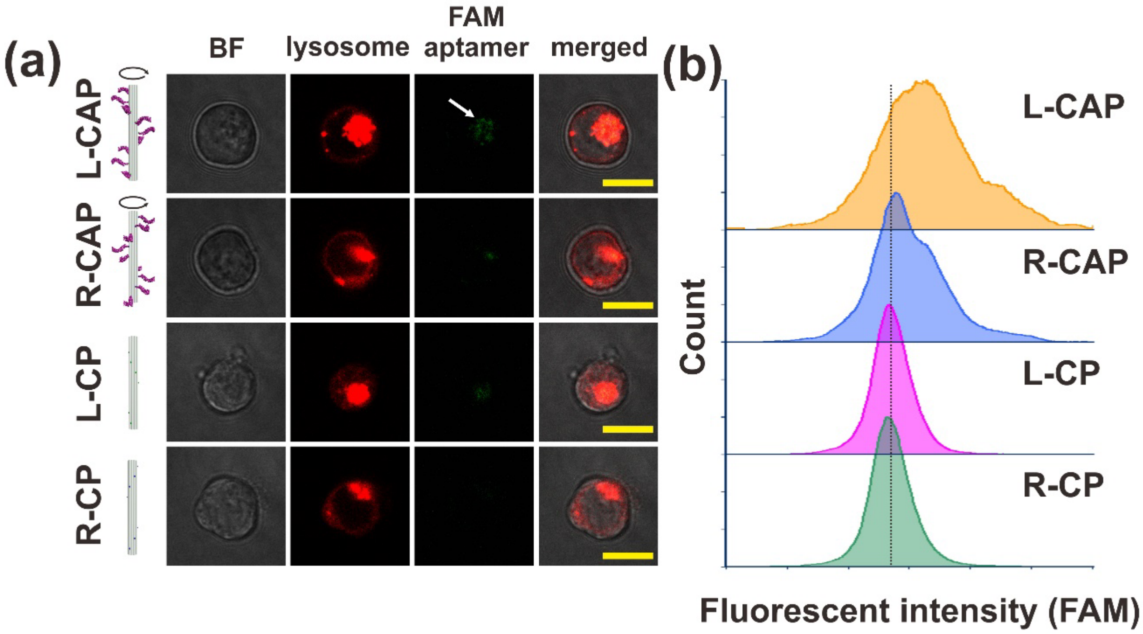

Figure S16. Confocal and flow cytometry analysis of K1 cells with and without chiral aptamer patterns showing the differential internalization at 2-hour incubation.

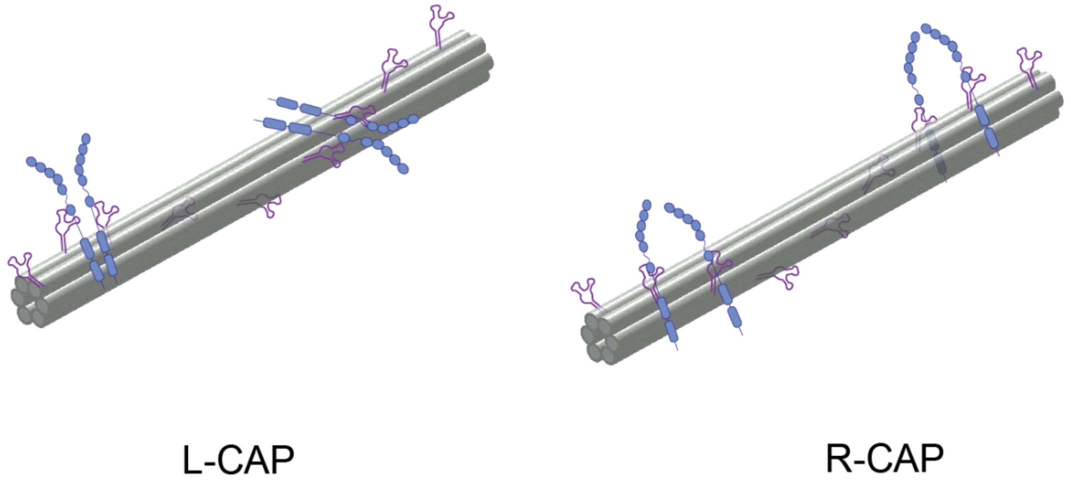

Figure S17: Proposed mechanism of enantioselective binding and internalization of L-CAP. We hypothesize that the orientation of the aptamer in L-handed arrangement allows binding to CD117 in a conformation that induces CD117 dimerization. Multiple such dimerization events eventually result in subsequent internalization of the CAPs. R-handed arranged however only allows binding of aptamer to CD117 though in a conformation that doesn't energetically favor dimerization, thus resulting in no internalization. DNA tube is shown in grey, Aptamer are shown in purple and CD117 protein monomers are shown in blue.

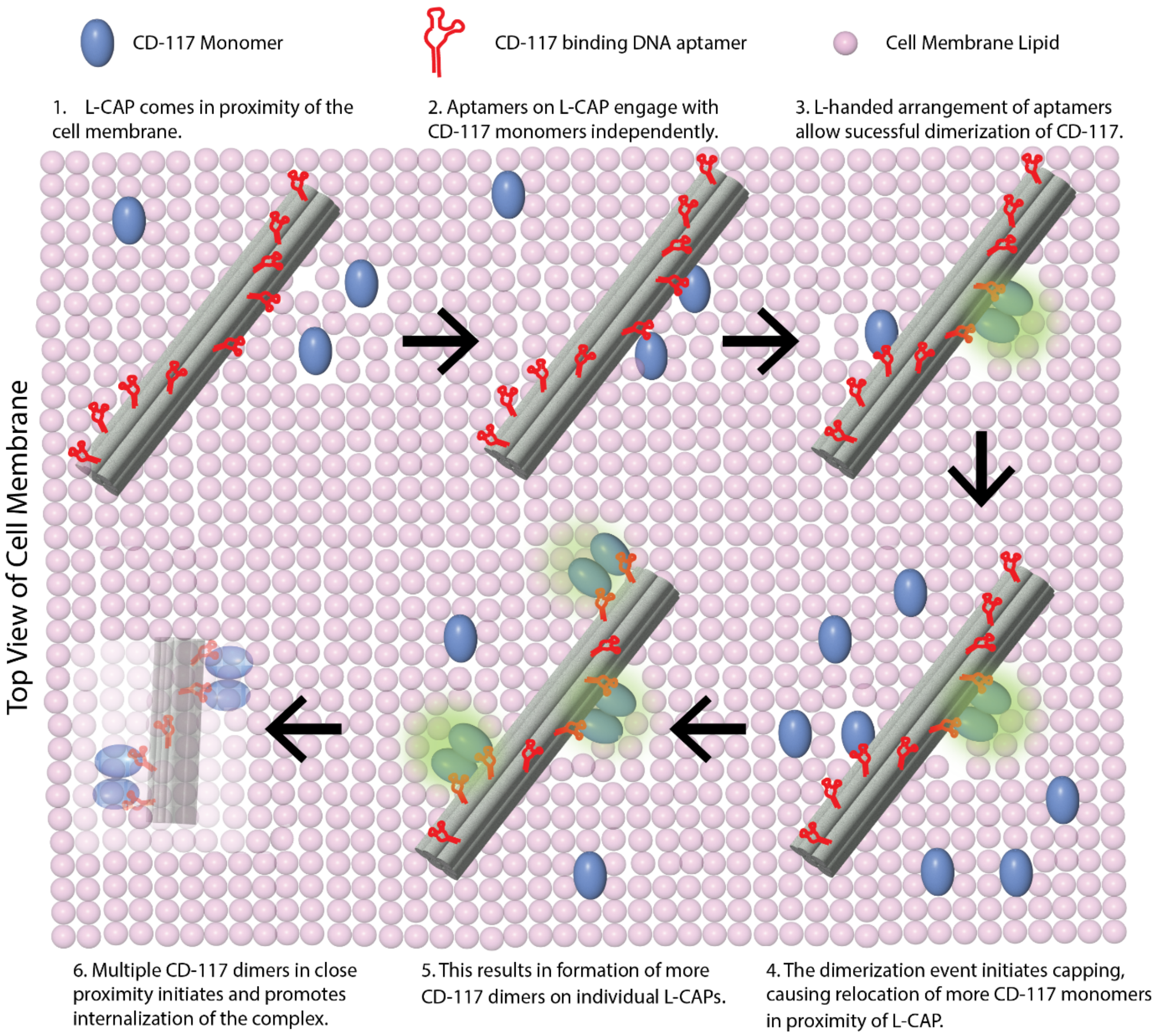

Figure S18: A schematic illustration of the proposed molecular events that facilitate the internalization of L-CAP. L-handed aptamer arrangement promotes CD117 dimerization, which drives receptor clustering and monomer recruitment, causing multiple dimers to form, thus promoting complex internalization.

Table S1. Staple DNA sequences for the formation of DNA tube containing L-handed docking sites.

Oligo1	ATGAGTATACGCTCGCCC
Oligo2	CGTAACCGATTGACTGCT
Oligo3	GAGGATTGCAAATGAAAA
Oligo4	AATATATTTTAGGCGTGCC
Oligo5	ACAGTGCCCGTATTGCGAA
Oligo6	GAAAGGCCGGAGACAGTCA
Oligo7	AAAGAAATTAACAATAGATAA
Oligo8	ACGAGAATGACCATAGACTGG
Oligo9	GGGATAGTTTGCTAAACAACT
Oligo10	TCAGGTCACTAGCATTCCCAG
Oligo11	AGGAAGGTTATATTACCGCCAG
Oligo12	CACCGCCTGCAGCCGCGCTTAA
Oligo13	CTCAAATATCATAAACAGGAGG
Oligo14	GCCCTAAAACAAGAAGATAAAA
Oligo15	GTGCGGCCCTGCTAATGAGTGA
Oligo16	TAAAGCCTGGGGTTTGGTGTAG
Oligo17	TTGTAGCAATATAGTAATAACA
Oligo18	ATATGGTTTACCAAAACTTTTTCC
Oligo19	CAGCACCCCAAAAACAGTAAGCAA
Oligo20	GAACAATCTAAAATATCTTTACGG
Oligo21	GCGAGAGCGCCAAAGACTTGGGTT
Oligo22	TGATAATCAGAAAAGGGAAGAGAA
Oligo23	ACCCTCGTAAAATTGCCTACGTCAA
Oligo24	AGGTGAGGGTTCAGACTGACAGAAT
Oligo25	CCAGAATAATACCAACGCTTTTACA
Oligo26	GATTAAGGTTGTAAACGGTAACGGT
Oligo27	GTAACACTCACATAACGTTACGAGG
Oligo28	GTTAATATTTTGTTTTCAAAAAAGC
Oligo29	TAAGTTTTAACGGGGTCTGAGTTAA
Oligo30	CGACATTTCAATAGAAAAGAATACAT
Oligo31	CAACAGCAGAGGCATTTTAATTTAGCG
Oligo32	ACCAGTAATAAAAGAATGGATTATTTAC
Oligo33	ACTTTCGCCATTGAGCTCGAATTCGTAA

Oligo34	AGAGATATAACACCCTGAACAAAGTCAG
Oligo35	AGGAGGCATTCCACTTTTGCGACCGATA
Oligo36	AGGGTCCGCGCATTAGAAAGGTCATTAA
Oligo37	ATATAACAATCATTACCGCTTGGTTAAA
Oligo38	ATATTCAATAGGCTTTTACCCAAACAGT
Oligo39	CATCAAAAATAATTCGATGTTCTTGTCAA
Oligo40	ATCCCTTATAAATCCGAAAATCCTGTTT
Oligo41	ATCGGCCAACGCGCTCCAGTCGGGAAAC
Oligo42	ATGCCTCAGGAATCACAATTCCACACAA
Oligo43	CCCTCAACAATACACTAAAACCGCGAAA
Oligo44	CCTACCATATCAAAATTATCGATTATAC
Oligo45	CGCAAATGCATTCTGCGAACGAGTAGAT
Oligo46	CGGATTTAGTAAATTTTGACCCAACCTA
Oligo47	CTTTTACGTAGATTCGTATTAAACAATT
Oligo48	GAACAAGAGTCCACGAATAGCCCGAGAT
Oligo49	GCAAACAACCCTAAAGGGAGCAGCAGTT
Oligo50	GCCAGCATAGAAGTCAACTAATAGATTA
Oligo51	GCGTAAGAATACGTATTCTGGCCAACAG
Oligo52	GGTGGCAAATAACCAAGCAAAGGTATGC
Oligo53	GTCCACGCTGGTTTCCCTTCACCGCCTG
Oligo54	TAGGGCGGGCTGTCAACAAGCAAAAATC
Oligo55	TATTAAGCCCCGATGGGAATTAGAGCCA
Oligo56	TCCCCGGGTACCCAAGCTACGTGGTGCT
Oligo57	TTGAATCGGCTGACCTGTAAGCAACTCG
Oligo58	TTTTCACCAGTGAGAGAGGCGGTTTGCG
Oligo59	AACGGTACGCCAGAATCCGAGTAAAAGAG
Oligo60	CGATCTAGGAAGTTTCCATGCTTATCGGA
Oligo61	AAACGCAAGCCGCCACACCACCAGAGCCGC
Oligo62	AAACTATCGGCCTTGCTGGTAATTTATCAA
Oligo63	ATATTTCCAGAATCTTAAGAGCAAATATCAG
Oligo64	GAGTTTCCTTAGAAACCAATCAATAATAGAA
Oligo65	GCCCGAACGTGACAACTTTCAGGTCAGAAAT
Oligo66	GTAGCCAGCTTTCATCAACATTTTGGTTTAA
Oligo67	TGCGGAAAGAGGGGATAAAAACCAAAATAGC
Oligo68	ACAACAAAGCTGCTCATTCAGTGATACTTTTG

Oligo69	AGCCTAGGGGGAGGGAAGGTAAATATTGAAAA
Oligo70	AGTATAAAGCCGTTACCAGAAGGAAACCGAGG
Oligo71	ATATATCCTGAGCCTAAGAAAAGTAAGCATGA
Oligo72	CACCAGCTCGCCATGAATGGCTATTAGTCTTT
Oligo73	CTTAAGACAAAAAATGCTTACCTTTCGGGCGC
Oligo74	GATTGAAGGAGAAAGTTTCAATTTCAGATGAT
Oligo75	GCTCACTGCCCGCAAATGTGAGCGAGGATAGG
Oligo76	GGATCAAGCCCACGCCTGTTGATACGCCCACG
Oligo77	TCACGGCCCACGACTTAAGTGGCTTCTATCGC
Oligo78	TCAGCGGAACAGTTAATGCCCCCTGCCTATTT
Oligo79	TGGAAATACCTACATTTTGACGCGAATATCCA
Oligo80	GAATTCTAAGTCAGGAGGGTTGTAGAAGAGTGA
Oligo81	AATCAATAAACATAGCAATAGCTATCTTACCGTT
Oligo82	ACGAGGGTAGCAATTCATGAAAGTTTTCTAATCC
Oligo83	ATAATTAAAGCCAGAATGACGATTAGAACCGAAT
Oligo84	TATATTGATCTTCTTGATATATCTGGTCAGTTGG
Oligo85	AAACGAAATGCCACAGACTTTCGGCTACCGTCACC
Oligo86	AACTAAAAGTTGATTCCCACTAAAACCGAAGGCTA
Oligo87	AAGATTACGCCTGTTTATCGCATGTAAAGTAATTC
Oligo88	AGAGCATTAGCAAAAAATCATAATAGTACTGAAAA
Oligo89	ATAGCAGCACCGACCCATCACCAGTAGCTACCAGG
Oligo90	ATAGCGTCGTTTTAGAAGCCCGGACGTTGAATTAC
Oligo91	ATCTACATCTATCAGGGCGATATATAACGTACGGT
Oligo92	ATTTACGACAAGAAGCTAATGACGACGAAAGAGAA
Oligo93	CAAAGTACAACGGAAGACGGTATGCTTTTGCGTAA
Oligo94	CAAGTTTCAATGAACCATTAGGCCATTTTTAGAGC
Oligo95	CACGTTGAAAGGAACCAAAAATTCAGGTAGAAAGA
Oligo96	CAGAAACGAGCGTCAACAGCCATATTATAGATAAT
Oligo97	CAGAACCCCTCAGAGAACCGCGCCATCTGGTCATA
Oligo98	CAGGCAATGGGAAGCAGCTGGGACTCCAGAGAGTC
Oligo99	CATCAATTAATGGAAGGGTTCCTGGCAATAGGTCT
Oligo100	CATTGACCATTAGACTATATTTTCATACCTTCAAA
Oligo101	CCAAGTATTTTGCACGACTTGATTTAGGTAGGGCT
Oligo102	CCCTCAGAGCCATAGGGGAACCAGAGCCCGTACTC
Oligo103	CTTATGCTGGGCTTAGAAACAAACGTAAGAACCGG

Oligo104	GAAACAAAATTACCCGCGCAGCAATAACACAGTAC
Oligo105	GAGAGAAAGAATTAACTGATTGGAAAGCCGTCACC
Oligo106	GAGCCACTGAGACTTGCTCAGACCATTAACCATCG
Oligo107	GCACTAAATTAGACTATTTTTTAAAAATACTTGCA
Oligo108	TAACCACCACACCCACAGTGCCACGCGGAACGAAC
Oligo109	TATTCGGAACCATCCGATAGTGTGAATTATCGGTT
Oligo110	TCGATGAAACGACGGCCAGATCTGTGCTGCAAGGC
Oligo111	TGGAGCAAACAAAATATTAAAGAACGTGCGAAAGG
Oligo112	TTAGTTATCATTCCGGCCCACTCGAAATCCGAGAG
Oligo113	TTCAAGCCACCCTCCACCCTCGGCCTTGCCGCCAC
Oligo114	TTCATCAGCAGATAAATATCGCCAATACTCAGAAA
Oligo115	TTGACGGCTGAAACAGAAGGATACCGCCACCCTCA
Oligo116	TTTACCAAAGAGCAAACTAATGTTGAGAAACGAAC
Oligo117	AATCAGAGCGGGAGCAACCCTCAATCACATTTGAGA
Oligo118	AATAAACACCGCATGATTAAGACTCCTTACCCAACAAA
Oligo119	ATTCAGGACAGATGAACCTTCATCAAGAGTTATATAAA
Oligo120	CAAAATCAATCGTCTGAGGGAATACCAAGTTTTTAGGA
Oligo121	CCTTACATAAGAATTACCTTTTTTAATACCGACCGTGT
Oligo122	CCTTACCAGGAACAAACGGCGGTGCATCTGCCAAATTC
Oligo123	TAACGGACCAGTCAGAAAGACGTCAGAATCAGGTCGGC
Oligo124	TATCAGCTCTCCAAACCCATGAAATATTCATTGGTATG
Oligo125	AGGTGAATTATCACCGGCGAACGTGGCGAGACGGGTAAC
Oligo126	CCAATAGGAATTCATGCGCATGCATTTCCTGATTGGCCC
Oligo127	CGAGTGCGCCGCAGTACACCTCATAGTTAGACGAGCAGG
Oligo128	CGGAGCGATTATACCAAGACTCATCGAATTTTGTCGTCT
Oligo129	GAGTTAAGCGAAAGACAGCACCCAAATCCCAGAACTATT
Oligo130	GATAAATTTTAGTAGCTTATCTTTTGAAGCCTTTCCTTT
Oligo131	GGCTTGCGGATTGCATCAACTTTGCTGATTAAATGTGAT
Oligo132	TACTACAGGCAAACAGGTTGATAAGAGGTCTGGCGTTGT
Oligo133	TTCTGAAATAATCCACAATTCTATTAATACAAAATTGAG
Oligo134	AATTACGCGAGGCGTTTTGATTTTTTAACATATAAAATTC
Oligo135	AAGTGTTTTTATTAGATTAAGACGCTGAGCCAATCGCAGAA
Oligo136	AAAATACACAAAGATTAAGAGATTCGAGCACATTCACACTAT
Oligo137	ACTTTGAAAGAACCGTTCTAGCTGATAATCAATACAATGCCT
Oligo138	AGGGCGACTTCGCTACGCCAGCTTGACAATGTCCCGGCACCG

Oligo139	AGGTTATAAGGGAAGAAAGCGCAAGAACGGGTAAACATCCTA
Oligo140	ATACGTAAGAGGCAAAAGAATCATAAGGGCTAAACGGGTAAA
Oligo141	ATCAAAATCACCATAGAGGCTTTATCGGCATTTTCTTTCATA
Oligo142	ATTTGGGGCGCGAGGTAGCATTACTACGTTAATAATTTAGGA
Oligo143	CAACTCGCTGAGGCTTGTAGTAAATGATTTTAAGAACAATGA
Oligo144	CATTTAAGAGTAACATTATCATTTTGAGTCCAAAATTAATTA
Oligo145	CCTGATTGCTTTACGGCACAGACGAATATACAGTAGGATTCG
Oligo146	CTTCTTCTCGTTTGAGGGGACGACGACAGCTTTCCGCCAAAA
Oligo147	GAAAAGGCGAATTTTAACAACGCCATACCGACAAAAGCGGGA
Oligo148	GACTTGACAAGGCCGATTAGCGTATTTATCCCAATGACACCA
Oligo149	GCCCCCTTTAGCGTGATATAAGGGGTTTCCTCAAGATGAAAG
Oligo150	GTCACAACAACCGATTGAGAAACGTCACGCTAAGTTTATTTT
Oligo151	GTCCTGAAGCATGTATCATTCAAAGGAGTTAACCTCCGGCTT
Oligo152	TAATTGTTCTTAAACAAAATAAATCCTCCAGGAGTGTGCCTT
Oligo153	TATAAAGACATGTACGGGAGGCGGTATTGCAAATCATTTTCA
Oligo154	TGAAAACACAATTTAAAACAGAACGTCAACGGAAAAAAAGGG
Oligo155	TTCCAGAGGACTAATACGAAGGCTAATCAGTAGCGTAGCGCG
Oligo156	TTTCACATCAAGAAAAATATGCGTTGAATCGCCATATATTCA
Oligo157	TTTTCGACGAAGTGCCGTCGAGTATCACACCACCGACCGCCA
Oligo158	CTTAGCCGGAACGAGGCGCGATTTGTCAAGTTTTTTGGGGTCG
Oligo159	CAGGGATTTTTGCGGATGGCTTAGACATGTTTTAAATTTGAGAGG
Oligo160	CGTAAAATTAACGTCAGATAATTTACAATGATTGTTCATATTCCT
Oligo161	TAACGTGCTTTCTTATCAAAATCAGTGTAGCGGTCACGCTGCGCG
Oligo162	TAATTGAATACAAAAAGCCTGAAGGCGTTCTGACCTAAATTTAAT
Oligo163	TTTTTGTTAAATCAGCTCATTTGGACGGGCAACTAAATTGTAAAC
Oligo164	TGAAATTGTTATCCGCGAATCTATTAACAGGGAAATCATTTCTCCG
Oligo165	TGTCCAGCAGAACGGTTGCTACCGCACTTCGTAGGTATATGTGAAC
Oligo166	ATATTGAGTTTCGTCACACTGGCTCATTATAACAACATTATTAGCTTT
Oligo167	CTGATAAATTGTGTACGTGAACCATCATAAAATGTTTAGTTACCCTCG
Oligo168	GAGAGACGATGCAACGTTTTTGACGGAACAAAGAAACCACCAATCAG A
Oligo169	AACCTCCCCCAGCTATGTTCAAAATAATATAATTGAGCGCTAGAAACA A
Oligo170	AACGGAATATTACGCAGTATTAGCGTTTCTCCCTCAGCCCGGCCACC CT

Oligo171	CACCACCCTCAGAGATATTCACAAACGCTAGTTTAGTACCGCATTTTCA
Oligo172	CGGAACTTATGTTAGCAAACGTAGAAAAACGCAAACCAAATAAAATAGC
Oligo173	CGGGAGAACATTATCTCCTTTCAGGATTAGACCGGTGTTTAGTACATTT
Oligo174	CTCAGCAAGGCCGCAGACAGCAACTACAAATAGGAAAAAAAGATTTTTT
Oligo175	GCGAACCAGAGAGTATCCAATATTAAGCAATTTAATCATTGTGGGAAGA
Oligo176	GGGATTAGGCTGCGCAACTGTAGCGCCACCAGCCAGTATCGGGGCGCAT
Oligo177	GTAGCTATTTTTGCTAGCTCAAGCTTAATTAATTGGACCCTGAAGCCTC
Oligo178	TAACCCCTCCTTAGCGTAATGGTAACAACCCGTCGGATTCTCCGTGGGC
Oligo179	TCAAAAAGCAAAGCCCTGACGGAGATGGTTTAATTTCAACTATAAATAA
Oligo180	TCACGACTTGGGTAATTACGCGGCGATCGGTGCGGGGGGTGCCGGAAAC
Oligo181	CAATAGCAACTAAGAACTAGAAATTCTTACCAAAAAAAAAAAAAAA
Oligo182	AATTTGAGTGATCAGTGAGGCCACCTGAGAAAAAAAAAAAAAAA
Oligo183	TGAAGAAACGCCAGTTAAAGAACTGGGAATCATAAAAAAAAAAAAAAA
Oligo184	TGATGGGCATAAGAGTCAATAGTGAATCTCGTTAGAAAAAAAAAAAAAAA
Oligo185	GTCTGGAAATGCTGGACCTGCTCCATGTTAAAAAAAAAAAAAAAA
Oligo186	CGGAACCTATACCCACAAGAATAGTGCCTTGAGTAAAAAAAAAAAAAAAA
Oligo187	CATAGTGACGACGGTAATAGCCCAAATATCATCGCAAAAAAAAAAAAAA
Oligo188	TAATAGCTCCAAAAGGAACTGGTAAAAAAAAAAAAAAAAA
Oligo189	AAAGAATAAAGCTAAATCGGTCTGGCCTTCCTAAAAAAAAAAAAAAA
Oligo190	ACCAAAAAGCCTTTATTTCAATTCGCATTAAAAAAAAAAAAAAAAA
Oligo191	GCCCTAAGCGTCATACATGGCTTTTGATGAAAAAAAAAAAAAAA
Oligo192	ACATAAAGGTGTTAATTTCATCTTAAATAAGAAAAAAAAAAAAAAA

Oligo193	ATATTAGACTGCCAAGCTTTCTGGTTGTGAACGCAAAAAAAAAAAAAAA
Oligo194	TGACGGTGTACAGACCAGGAACCGAACTGACCAAAAAAAAAAAAAAAA
Oligo195	TGGTTGCTTTGACGAGCACGTAAAAAAAAAAAAAAAA
Oligo196	AATAATAATAATAATCAAAAGAAGTTTTGCCTCGTCATTACC
Oligo197	AATAATAATAATAATGAGAGGCTTAACAACTAAAGGAATAAGTGAGAAAT
Oligo198	AATAATAATAATAATCTGAATTTACCGAAGCCCTTTTTCGAGCCAGT
Oligo199	AATAATAATAATAATCAGGAGGTTGAGGCAGGTCAGGAAAGCGCAGTCT
Oligo200	AATAATAATAATAATCGCCAGCATGATAGCCGAACAAAAACGCT
Oligo201	AATAATAATAATAATAAAATTTTTAGAACCCTCATATATAATCTTG
Oligo202	AATAATAATAATAATGGTTTGAAATGGAAACAGTGCTTCTGTAAA
Oligo203	AATAATAATAATAATGAGTAATGTGTAGGTAAAGAAAAACGCAAGGATA
Oligo204	AATAATAATAATAATTCGTCGCTATTAATTAAGATATAATTTTCACATC
Oligo205	AATAATAATAATAATAATCACCAATTAATGCCGGTCAATCATATGT
Oligo206	AATAATAATAATAATTCCTTGAAAACATAGCGATAGCTAAATAA
Oligo207	AATAATAATAATAATAGGTGCCGTAAAGCACTAATAGAAAGGTTG

Table S2. Staple DNA sequences for the formation of DNA tube containing R-handed docking sites.

Oligo1	GGTTGCTCCTTTTGTTTAA
Oligo2	AATGCCCAGTAACATACAGGA
Oligo3	CAATCATGTTGATATAAGCAA
Oligo4	TTCCAGTAAGCGTCTAAAGCC
Oligo5	TTGAGCCATTTGGGCGGCGAA
Oligo6	TTTGAGAATCGATGTTCCCAG
Oligo7	AAGAAGAACGTTATTAATTGAGTAAC
Oligo8	GATAGCGTCTCAAATTGCAACCCACT
Oligo9	TTTTTAACCCATAGTTTCAGCCTCAA
Oligo10	AAAAGGCATGGAATTGCGAATAATAATT
Oligo11	AGAAGGCAGCGAACATCATATACGCTCA
Oligo12	AGTTTGAGCTCCATATAACAGTTGATTC
Oligo13	ATGTGAGTGAATTCAACATTTAATTATA
Oligo14	ATTATCAGGAATTATCATCAGAAGATAG
Oligo15	CAGATGAGTAAAACGACTTTAGTATATT
Oligo16	CATTTGGTAGATACGCTTCAAGGCTGTA
Oligo17	CTGAGAGCTGATGCGAATCATAAAGTTT
Oligo18	CTGCTCATTCAGCCCAAGGATAAAGTAA
Oligo19	GAAGCCCAATAATATCCAGAGCTAAACA
Oligo20	GAATTTCAAAGGAGAGTTTCGTCTTGCT
Oligo21	GAGTTAATAGGGTAATTGAGCGCTAATA
Oligo22	GGTTGTTAGTAAATCCAAGCGAAAAGAA
Oligo23	TAGGGCGCCAAGAATTTCATCCATTTGC
Oligo24	TTCTGAATAATGGAAGGGTCTATCAATA
Oligo25	TTTTCTAAGTGAGAAATCGGAACAAAGT
Oligo26	GCTGACGGTGTACAGACCGGGAACCGAACTGAC
Oligo27	GTCTGAACGACGGCCAGATCTGTGCTGCAAGGC
Oligo28	AAACAACGGAACAACTAAAAGTCGAGGTTATCATC
Oligo29	AAGAAAACAAAATTAACAATTTCATTTGTCAATAT
Oligo30	AAGATTATACATAACGCCACATTTACAGGTAGAAA
Oligo31	AAGCCCCTAAAACTCAAGAGAGATCTACACGTCAA
Oligo32	AATAGTAGCCCGAATATAGTCCAGGACGGTGAATT
Oligo33	AATTGCAACAGGAAATTTACATTGGCGGATGAGGA

Oligo34	ACAGAATCAAGTGCCGAGCAAGGCCGGATCGAGAG
Oligo35	ACAGTAGGGCTTCAGAAGCCTGTTTAGTCTCCCGA
Oligo36	ACCCAGCATAAGTCCTGAAACATCGACGACAATAA
Oligo37	ACCTTATATTGGGCCGAGAAATCAACGTAAGAACC
Oligo38	ACGAGGCAAATTGTCAAGTTTTTTGGGGCGAGAGG
Oligo39	AGAATAAACAAATTACGCGAGATTAGTTGCTATAA
Oligo40	ATATTAGACTGCCAAGCTTTCTGGTTGTGAACGCC
Oligo41	ATCAGGTCATTGAATATTAAAGAACGTGCGAAAGG
Oligo42	ATTCTGCGAACGAGAAATGGTCAATAATAAATATC
Oligo43	CAAAATTTCATACAAGTAGTAAAAAGGTATATTTT
Oligo44	CAATCAATCCTAATTTTATCAGTTCAGCCAAAAGG
Oligo45	CAGCATTCACCACCCCCTCAGCCGGAACTGCCATC
Oligo46	CAGGGAGCGATATAACAACAAGATACCGTCGAGGT
Oligo47	CATTTTCGGTAATAAGTTTCCTTCTGAATTTACCG
Oligo48	CCAATTGAGAATCGTAATAAGAGAATAACAGGGAG
Oligo49	CCACCTAGAAGGCAGGTCAGAAAAGCGCAGTCTTT
Oligo50	CCGCCACCAGAATGGAGCCTCCCTCAGACCGCCAC
Oligo51	CCTGATTGATTGTTTGGATTTCTGGCAACATAGGT
Oligo52	CCTTACGCAGAACAAACGGCGGTGCATCTGCCACG
Oligo53	CGGGTATCTGTCTTTCCTTATGCCAAGAAAAATAA
Oligo54	CGTTTACGTAAGAGTCAACTACAGTTGAAAAACGA
Oligo55	CTTTTGCCGATAAAAACCAAATCAAAGGAATTACG
Oligo56	GAAAAAATAAGTTTATCCGACAAATATTATGATAT
Oligo57	GAACAAGTTATCCTAGCCTTAGAGCCAGCCATATT
Oligo58	GAAGGTAGCGACATATCAATACACGGAAATACATA
Oligo59	GAGAAGGGCAAGCCTCAGAACCCTTGATCCGCCGC
Oligo60	GAGGCGAGAATACCAATAACGCAGTACCTTAACGT
Oligo61	GCCCACCACCAGAGATTCACAAACAACCTAGAACC
Oligo62	GCTCAACTGGAAGTTTCATCTAAAACCGAGCTATT
Oligo63	GGATATTGCATAGGCAGTTCAGGAATCGTAGACTG
Oligo64	GGATTCAGTTTGACCCCCAGCAGATTTGGCCGTAA
Oligo65	GGGAAGCAACAAAGTCAGATTGGAAAGCAATTAGA
Oligo66	GTAGCGCCGTAATCCCAATGAAAATCACTTAGAGC
Oligo67	TAACAACAAAGCCAGCGTTATACACCGGTAAATAA
Oligo68	TACACTAACCTAAATTCCATTACTAAAGGACAGCA

Oligo69	TGGCATGATAATAAACCAGAAAGTAAGCTCTTACC
Oligo70	TTCATCAACATTTTGGTTTAACCAATAGGAATTCA
Oligo71	ACTAACGTACCAGTAGAAGCATGACCATCTTTAAACTG
Oligo72	GGTGATACAGAGCGTCAAACATATTAAAACGTAAAAGAAC
Oligo73	TAAAGTACATTTTCAATCAAGGCGTTTTTTATCCGAATTT
Oligo74	TTTTCATTCATCGGTAGGTGTGCGGATATTAGCGGATGAA
Oligo75	GGCTTAAATCAGGTCTTACTTGCGGAATTTTTACAAAAGG
Oligo76	AAACGCAATTAAGACTCCTCCTCAGAGCGACAGGAGGTTGGG
Oligo77	AAAGATTTTCAGGTTTTTACATCAGATTCACCAGTTGACCTG
Oligo78	ACAGTAAGATTCGCCTTTTAGGCAGAGGATTCTGTCCAGAAT
Oligo79	ACCACCGGAACCAAACTTTTTCACTTATTAGCGTTCAGAGCC
Oligo80	AGGCATACAGACGAAAAAGAACCCAAATGTCGAAATCCGCGA
Oligo81	AGGGCGACTTCGCTACGCCAGAGTGACAATGTCCCGGCACCG
Oligo82	ATCAAAATAAATTTGTTAATATTTTGTTACAGGAAAAGCCAG
Oligo83	ATCAGCTCATTTGGACGGGCAACTAAATTGTAAACTTGTTAA
Oligo84	CAAAAGGAATATTGACGGATAGCAGCACGTCAGCGCCAAAGA
Oligo85	CAATAAAAAGCAATAAAGCGTCTGGCCTGATTCTCCGTGGTC
Oligo86	CCAATCATAAAGTAGGCCCACTAAATTAATGCCGGTTCAACC
Oligo87	CCATTAAACCCTAAAGGGAGCAGGCTGAGACTCGGAACAGTT
Oligo88	CCTGCGATACGTGAACCATCAGTTTTGCCAGAGACTAACCCT
Oligo89	CGAGCTGGCATTAAAACCAGAGAGTACCTTTAATGGCCTCAG
Oligo90	CGTATGTTAACCACCACACCCCGGTCAGTATTATCAAAACAT
Oligo91	CTTCTATGGGTTTGAGGGGACGACGACAGCTTTCCGCCAAAA
Oligo92	GAAAATCCATACCCTGACTATAGACTTCACCACATCAACACT
Oligo93	GAACCTGTTTAGCTGGCATCAATATCTACGTTAATGATTTAG
Oligo94	GATAGCAATAGCTAAGATAGCCGATAAAGTACCGATAATGCA
Oligo95	GATTCATATGCAGAAGAGGAAAAATGTTTCATAAAAGCCGGA
Oligo96	GCCAGCAAACCATCGTACCAGATGAAACGATTTTTCATATGG
Oligo97	GCCCCTGCGATATAAGTATAGTTTAGTAGCCGCCACTCAGAG
Oligo98	GCCTGATGCAGACGATACTGCGACGTAACGATCTATAAAATA
Oligo99	GGCACCAAAACACTCATCTCAATCATAAAGTGCCACTACGAA
Oligo100	GTAAATCTGAAAACATAGCATCCAATCGCTAACCTTGCTTCT
Oligo101	GTAATAACATCAACTTTTTTAATAGAGTCTGTCCATTGATTA
Oligo102	GTGTACTAGGGATAATTAGGAAGTGCCGAACGTCAAGTAGCG
Oligo103	TAAGAAAGGAAACCATTTGCCCAATCCAAATAACAACTATTA

Oligo104	TAATCCTATCAGATATTTAGAGTATTAAGAGAAACAAGTTAC
Oligo105	TATCCCATAATCGGTAAACCAAAAGGAGTTTTAACCTCCGGC
Oligo106	TATGATAAGAGGGTTCTATCAGGGCGATCGGTGTCATGTTTT
Oligo107	TCCGCGAATCTATTAACAGGGAAATCATATAAATCCGAAAAT
Oligo108	TGCGTAGTTTGAGGGATGGCACCACCAGCTGAGAATCCCTTA
Oligo109	TTAGGGTAAGGGAAGAAAGCGAGTACCGCACTCTTTAGAAAC
Oligo110	TTGCTTTATTATTCATTTCTACCAGTATGCCAACATGTAAGA
Oligo111	CAACTTTGAAAGACTTCCATGTTACTTTATTCATTGAATGCCAGGGGT
Oligo112	AAATATGTTAGAGCTTAATTGGTCATTTAGGTCAGTCGGTTGGAATTAG
Oligo113	AACTTGCCTGAGTATTGAAAGGAATTCCGAACAAAAAACAGTTTAATTA
Oligo114	ACAACATACAATAGTACAATTCAAGCCGCCCAATAACTATATAAGAACG
Oligo115	ACAACGGGATTATAGAATTTTGTCGTCTATTCCACCAGCAGCAGGCTT G
Oligo116	AGAATGGCGATTGGCGCCACCCAGGAGGCCCGGAACATTTTCTCAGA CT
Oligo117	AGCACTAATAGAAATTTCAACGTACCGTACGCCTGGACAATGTTCGGT C
Oligo118	AGCATAACCTGTAATACTTAATTCGCATATAATTCGATGTTCTTGTCAA
Oligo119	AGGTTATTAATACACGTAAGACTGATAGCCTAGAACCTACCAAAGAAAT
Oligo120	CAATTCGACAACTCAGTATTAAGAAATATATCAAAATTATTTCATATAC
Oligo121	CATAAAGTGTTAGCTTTATCCAGTTACAGCGTCTTAGAGCAAAAGAATT
Oligo122	CCTCAGCTTCCAGAAGGAAGTACGAAAGAGTTGCCTTTAGCGGGTCAT A
Oligo123	CGAGAAAACTTTTGCCTTGGGTTATATAGCAAGCAAATCAAGAAATCG A
Oligo124	CGCAGTAGTGGCAACATATTCAAAATCAAACCGCCCCGTACTCTCAGA G
Oligo125	CGTAACCGATTGACTGCTGAATTCTAAGTCAGGAGGATTGTAATCAGA A
Oligo126	CGTAAGCCAGGCTACAGAGGCTTTGAGGAAACGGGAAGTTTTCTGTAT G
Oligo127	CGTGGCGCACCCTGGCATTAGCTAACGAAAATAAACAAAGTTCGGAAT A
Oligo128	CTTAGATTCAAAATGTGTAGCGGTCACGCTGCGCGCAATAGTGAATTT A
Oligo129	CTTGCGCAATGAAATGTCACATCAACCGATTTCAAATATATTTTAGTTA

Oligo130	GCGTTTTCAAACTCTACTAATGGCAAGGCAACTTTAATCATTTTGGGAA
Oligo131	GGGATCGGAGGGTAGCAACTTACCCAAACACCAGAACGAGAAAAGCGGA
Oligo132	GGGATTAGGCTGCGCAACTGTAGCGCCACCAGCCAGTATCGGGGCGCAT
Oligo133	GTTCTAGGAAAGGCCGGAGACAAAGATTGAACCCTTTCAACGAAAAACA
Oligo134	GTTTTGAGAATCTTGCGCCTGTTACGAGCAGAGATAACCCACGAAACAA
Oligo135	TAACCCCTCCTTAGCGTAATGGTAACAACCCGTCGTCCTGTAGCCAGCT
Oligo136	TCACGACTTGGGTAATTACGCGGCGATCGGTGCGGCCGGTGCCGGAAAC
Oligo137	TCGGAACTCACCCTAGACAGCAACTACAAACACTGCCTTTAATCCAAAA
Oligo138	TGAGCGAGGATAGGTCACGGCCCACGACTGCGCATGCATTTCCTGATTG
Oligo139	TTAAGTGGCTTCTATCGCACTTTCGCCATTGAGCTCGAATTCATTGTTA
Oligo140	TTATGACAGCTAAAGATTAGACCGGAAGAATTCGAATTTCGCTAGATTT
Oligo141	TTTACTTAAAAAAGAAACGCAAAGACACGAAAATTTGTTTAAAGAATAA
Oligo142	AATAATAATAATAATAGTATTAAGCCCCGATCAGTAGCACCATTA
Oligo143	AATAATAATAATAATAACCTATGCTAACGGGGTCAGTGCCTTGCCTGCCTATTTCGG
Oligo144	AATAATAATAATAATACAGCTTCCATCGCCAGTACACCTCATAGTTAGAAACTTTGC
Oligo145	AATAATAATAATAATGGCTCATTAGAACAACATTATAA
Oligo146	AATAATAATAATAATGCATAACTTAAAGGCCGCTGAGTAGTAAGCGATTTTAAGAAC
Oligo147	AATAATAATAATAATCCTTTATCATATATATAAGAGCTGAATATAATGTTAAATCAA
Oligo148	AATAATAATAATAATGGCGTTATGACCTAGTATTCTTACCGCGTTTTTAT
Oligo149	AATAATAATAATAATATGCAATAAAATTGCGGGAGAAG
Oligo150	AATAATAATAATAATAATGGTTTGCGTGTGAAATCATAATTACTA
Oligo151	AATAATAATAATAATTGTAGGTAGTCAAATCACCCGGTAATCGAAGCCTGAGTAATG

Oligo152	AATAATAATAATAATATTTCATCAAGACAGTAAATGACTACCTCGGGCGC
Oligo153	AATAATAATAATAATTTGACGGCTGAAACGGTTTTGCCACCCT
Oligo154	GCCACCCCAATAGGTTGATGAGTGCCCGAAAAAAAAAAAAAAA
Oligo155	TTAAAGACTAATTACCTGAGCAAAAAAAAAAAAAAAAA
Oligo156	CATAACAGAAATTATTCATTAAAAAAAAAAAAAAAAAA
Oligo157	GATTAAGGTTGTAAGAGCAAAAGCATGTAAAAAAAAAAAAAAA
Oligo158	TCAGATGAACTGAAAGAAAGGCACCGACAAAAAAAAAAAAAAA
Oligo159	CAGGCAATGGGAAGCAGCTGGGACTCCAAAAGGCTAAAAAAAAAAAAAAA
Oligo160	TAACGTTGAAAATCTTGTATCGGAAAAAAAAAAAAAAA
Oligo161	TGAAAACACCAACGACGGGAGCAGCCTTATTATCAAGGGAGGAAAAAAAAAAAAAAA
Oligo162	GCATCAGCTTGCTTATAGTTGCGATAAATCCTCATATACATGAAAAAAAAAAAAAAA
Oligo163	GGTTTAATTTCAAATATGAATAAAAAAAAAAAAAAAAA
Oligo164	TCAAGAATGCCCCCTCAAATGAAATCAAGCCCTGATTGAGATAAAAAAAAAAAAAAA
Oligo165	GCCCCAGCACCCCGATGTAGGAACCTGAGAAAAAAAAAAAAAAA
Oligo166	GTGAGGACAGATGAACCTTCATCTTGACAACAAAGAAAAAAAAAAAAAAA
Oligo167	GAATCCTGTCGCTAACATAAAAATTACCAAACATCAAAAAAAAAAAAAAA
Oligo168	TAAGACGAAGGAGCTTTTGCGTTTGCCCGATGATGTCGCGCAAAAAAAAAAAAAAA

Table S3. The sequence of DNA attached to AuNP surface.

Name	Sequence
Linker strand-1	/5thiomc6-d/TTTTTTTTTTTTTTTTTTT
Linker strand-2	ATTATTATTATTATTTTTT/3thiomc3-d/
Spacer strand	/5thiomc6-d/TTTTT

References:

1. Yuzawa, S.; Opatowsky, Y.; Zhang, Z.; Mandiyan, V.; Lax, I.; Schlessinger, J., Structural basis for activation of the receptor tyrosine kinase KIT by stem cell factor. *Cell* **2007,** *130* (2), 323-34.
2. Zhang, Y.; Xiong, Y.; Yang, C.; Xiao, Y., 3dRNA/DNA: 3D Structure Prediction from RNA to DNA. *Journal of molecular biology* **2024,** *436* (17).
3. Honorato, R. V.; Koukos, P. I.; Jiménez-García, B.; Tsaregorodtsev, A.; Verlato, M.; Giachetti, A.; Rosato, A.; Bonvin, A. M. J. J., Structural Biology in the Clouds: The WeNMR-EOSC Ecosystem. *Frontiers in Molecular Biosciences* **2021,** *8*.
4. DeLano, W. L. The PyMOL Molecular Graphics System. http://www.pymol.org.
5. Douglas, S. M.; Marblestone, A. H.; Teerapittayanon, S.; Vazquez, A.; Church, G. M.; Shih, W. M., Rapid prototyping of 3D DNA-origami shapes with caDNAno. *Nucleic Acids Research* **2009,** *37* (15), 5001-5006.
6. Pettersen, E. F.; Goddard, T. D.; Huang, C. C.; Couch, G. S.; Greenblatt, D. M.; Meng, E. C.; Ferrin, T. E., UCSF Chimera?A visualization system for exploratory research and analysis. *Journal of Computational Chemistry* **2004,** *25* (13), 1605-1612.
7. Poppleton, E.; Romero, R.; Mallya, A.; Rovigatti, L.; Šulc, P., OxDNA.org: a public webserver for coarse-grained simulations of DNA and RNA nanostructures. *Nucleic Acids Research* **2021,** *49* (W1), W491-W498.
8. Poppleton, E.; Bohlin, J.; Matthies, M.; Sharma, S.; Zhang, F.; Šulc, P., Design, optimization and analysis of large DNA and RNA nanostructures through interactive visualization, editing and molecular simulation. *Nucleic Acids Res* **2020,** *48* (12), e72-e72.
9. Song, T.; Liang, H., Synchronized Assembly of Gold Nanoparticles Driven by a Dynamic DNA-Fueled Molecular Machine. *Journal of the American Chemical Society* **2012,** *134* (26), 10803-10806.
10. Song, T.; Xiao, S.; Yao, D.; Huang, F.; Hu, M.; Liang, H., An Efficient DNA-Fueled Molecular Machine for the Discrimination of Single-Base Changes. *Advanced Materials* **2014,** *26* (35), 6181-6185.
11. Dempsey, G. T., A User's Guide to Localization-Based Super-Resolution Fluorescence Imaging. In *Digital Microscopy*, 2013; pp 561-592.
12. Dempsey, G. T.; Bates, M.; Kowtoniuk, W. E.; Liu, D. R.; Tsien, R. Y.; Zhuang, X., Photoswitching Mechanism of Cyanine Dyes. *Journal of the American Chemical Society* **2009,** *131* (51), 18192-18193.
13. Rust, M. J.; Bates, M.; Zhuang, X., Sub-diffraction-limit imaging by stochastic optical reconstruction microscopy (STORM). *Nature Methods* **2006,** *3* (10), 793-796.
14. Dani, A.; Huang, B.; Bergan, J.; Dulac, C.; Zhuang, X., Superresolution Imaging of Chemical Synapses in the Brain. *Neuron* **2010,** *68* (5), 843-856.
15. Martin-Fernandez, M. L.; Tynan, C. J.; Webb, S. E. D., A 'pocket guide' to total internal reflection fluorescence. *Journal of Microscopy* **2013,** *252* (1), 16-22.
16. Axelrod, D., Total Internal Reflection Fluorescence Microscopy in Cell Biology. *Traffic* **2001,** *2* (11), 764-774.
17. Burnette, D. T.; Sengupta, P.; Dai, Y.; Lippincott-Schwartz, J.; Kachar, B., Bleaching/blinking assisted localization microscopy for superresolution imaging using standard fluorescent molecules. *Proceedings of the National Academy of Sciences* **2011,** *108* (52), 21081-21086.
18. Ovesný, M.; Křížek, P.; Borkovec, J.; Švindrych, Z.; Hagen, G. M., ThunderSTORM: a comprehensive ImageJ plug-in for PALM and STORM data analysis and super-resolution imaging. *Bioinformatics* **2014,** *30* (16), 2389-2390.
19. Dwivedy, A.; Baskaran, D.; Sharma, G.; Hong, W.; Gandavadi, D.; Krissanaprasit, A.; Han, J.; Liu, Y.; Zimmers, Z.; Mafokwane, T.; Hayah, I.; Chauhan, N.; Zheng, M.; Yao, S.; Fraser, K.; Decker, J. S.; Jin, X.; Wang, H.; Friedman, A. D.; Wang, X., Engineering Novel DNA Nanoarchitectures for Targeted Drug Delivery and Aptamer Mediated Apoptosis in Cancer Therapeutics. *Advanced Functional Materials* **2025,** *35* (22), 2425394.
20. Broudy, V. C.; Lin, N. L.; Bühring, H.-J. r.; Komatsu, N.; Kavanagh, T. J., Analysis of

c-kit Receptor Dimerization by Fluorescence Resonance Energy Transfer. *Blood* **1998,** *91* (3), 898-906.
21. Lennartsson, J.; Jelacic, T.; Linnekin, D.; Shivakrupa, R., Normal and Oncogenic Forms of the Receptor Tyrosine Kinase Kit. *Stem Cells* **2009,** *23* (1), 16-43.
22. Lennartsson, J.; Rönnstrand, L., Stem Cell Factor Receptor/c-Kit: From Basic Science to Clinical Implications. *Physiological Reviews* **2012,** *92* (4), 1619-1649.
23. Reber, L.; Da Silva, C. A.; Frossard, N., Stem cell factor and its receptor c-Kit as targets for inflammatory diseases. *European Journal of Pharmacology* **2006,** *533* (1-3), 327-340.
24. Le Gall, M.; Crépin, R.; Neiveyans, M.; Auclair, C.; Fan, Y.; Zhou, Y.; Marks, J. D.; Pèlegrin, A.; Poul, M.-A., Neutralization of KIT Oncogenic Signaling in Leukemia with Antibodies Targeting KIT Membrane Proximal Domain 5. *Molecular Cancer Therapeutics* **2015,** *14* (11), 2595-2605.
25. Kim, S. Y.; Kang, J. J.; Lee, H. H.; Kang, J. J.; Kim, B.; Kim, C.-G.; Park, T.-K.; Kang, H., Mechanism of activation of human c-KIT kinase by internal tandem duplications of the juxtamembrane domain and point mutations at aspartic acid 816. *Biochemical and Biophysical Research Communications* **2011,** *410* (2), 224-228.
26. Sheikh, E.; Tran, T.; Vranic, S.; Levy, A.; Bonfil, R. D., Role and Significance of c-KIT Receptor Tyrosine Kinase in Cancer: A Review. *Bosnian Journal of Basic Medical Sciences* **2022**.
27. Heo, S.-K.; Noh, E.-K.; Kim, J. Y.; Jeong, Y. K.; Jo, J.-C.; Choi, Y.; Koh, S.; Baek, J. H.; Min, Y. J.; Kim, H., Targeting c-KIT (CD117) by dasatinib and radotinib promotes acute myeloid leukemia cell death. *Scientific Reports* **2017,** *7* (1).
28. Czechowicz, A.; Palchaudhuri, R.; Scheck, A.; Hu, Y.; Hoggatt, J.; Saez, B.; Pang, W. W.; Mansour, M. K.; Tate, T. A.; Chan, Y. Y.; Walck, E.; Wernig, G.; Shizuru, J. A.; Winau, F.; Scadden, D. T.; Rossi, D. J., Selective hematopoietic stem cell ablation using CD117-antibody-drug-conjugates enables safe and effective transplantation with immunity preservation. *Nature communications* **2019,** *10* (1).
